# Comparative genomics of canine hemoglobin genes reveals primacy of beta subunit delta in adult carnivores

**DOI:** 10.1186/s12864-017-3513-0

**Published:** 2017-02-08

**Authors:** Sara Zaldívar-López, Jennie L. Rowell, Elise M. Fiala, Isain Zapata, C. Guillermo Couto, Carlos E. Alvarez

**Affiliations:** 1 Center for Molecular and Human Genetics, The Research Institute at Nationwide Children’s Hospital, Columbus, OH USA; 2 College of Veterinary Medicine, The Ohio State University, Columbus, OH USA; 3 College of Nursing, The Ohio State University, Columbus, OH USA; 4 College of Medicine, The Ohio State University, Columbus, OH USA; 5 Present affiliation: SZL, Departamento. de Genetica, Facultad de Veterinaria, Universidad de Cordoba, Cordoba, Spain; 6 Present affiliation: Center of Excellence in Critical and Complex Care, College of Nursing, The Ohio State University, Columbus, USA; 7 Present affiliation: Couto Veterinary Consultants, Hilliard, OH USA

**Keywords:** Canine, Hemoglobin, Globin, Genetics, Comparative genomics, HBH, HBD, HBB

## Abstract

**Background:**

The main function of hemoglobin (Hb) is to transport oxygen in the circulation. It is among the most highly studied proteins due to its roles in physiology and disease, and most of our understanding derives from comparative research. There is great diversity in Hb gene evolution in placental mammals, mostly in the repertoire and regulation of the β-globin subunits. Dogs are an ideal model in which to study Hb genes because: 1) they are members of Laurasiatheria, our closest relatives outside of Euarchontoglires (including primates, rodents and rabbits), 2) dog breeds are isolated populations with their own Hb-associated genetics and diseases, and 3) their high level of health care allows for development of biomedical investigation and translation.

**Results:**

We established that dogs have a complement of five α and five β-globin genes, all of which can be detected as spliced mRNA in adults. Strikingly, HBD, the allegedly-unnecessary adult β-globin protein in humans, is the primary adult β-globin in dogs and other carnivores; moreover, dogs have two active copies of the *HBD* gene. In contrast, the dominant adult β-globin of humans, *HBB*, has high sequence divergence and is expressed at markedly lower levels in dogs. We also showed that canine *HBD* and *HBB* genes are complex chimeras that resulted from multiple gene conversion events between them. Lastly, we showed that the strongest signal of evolutionary selection in a high-altitude breed, the Bernese Mountain Dog, lies in a haplotype block that spans the β-globin locus.

**Conclusions:**

We report the first molecular genetic characterization of Hb genes in dogs. We found important distinctions between adult β-globin expression in carnivores compared to other members of Laurasiatheria. Our findings are also likely to raise new questions about the significance of human *HBD*. The comparative genomics of dog hemoglobin genes sets the stage for diverse research and translation.

**Electronic supplementary material:**

The online version of this article (doi:10.1186/s12864-017-3513-0) contains supplementary material, which is available to authorized users.

## Background

Dog models are rapidly rising in diverse areas of biomedical research [1, 2]. Although they can be used experimentally, the advantages that are gaining interest are related to the facts that they are natural models of complex traits with epidemiology and extremely powerful translational genetics. Most investigators do not consider dogs an alternative to mice, but rather a complementary organism with many similarities to human research. However, dogs have the immense advantage of having approximately 400 isolated populations or breeds – each on the order of 100-fold genetically simpler than the dog or human population. As a result, canine investigations have begun yielding important new understanding of complex-genetic traits that have been difficult to study in humans, who have vastly greater heterogeneity. Examples of major successes include diverse morphological traits [3, 4], germ line risk of rare cancers [5, 6] and anxiety- and aggression-related behaviors [7]. In the last year, the first major genome wide association study of canine blood traits was published. Using 353 clinically healthy dogs, White, Boyko and colleagues found significant loci for alanine transferase, amylase, segmented neutrophils, urea nitrogen, glucose and mean corpuscular hemoglobin [8]. Yet, while canine genetics are exceptionally powerful, one major bottleneck is gene annotation.

Hemoglobin (Hb) is among the most highly studied proteins because of its central role in physiology and its alteration in hemoglobinopathies, some of which are very common (e.g., sickle-cell disease). Hb proteins were among the first to be characterized by structure and function, and comparative studies were among the earliest tools used by biochemists to try to understand normal and disease-mutant Hb in the 1950’s [9]. In the pre-genomics DNA era, β-globin genes were some of the earliest prototypes of gene duplication, gene/protein evolution, and tissue- and developmental-specific transcriptional regulation [10]. The gene for a human Hb subunit (*HBB*) from β-thalassemia patients was one of the earliest to be targeted in the nascent field of gene editing [11]. There is a wealth of knowledge on Hb gene regulation and protein function in humans, mice, and chickens [10]. However, despite fine comparative genetic studies of Hb genes in placental mammals and animals in general, there is a gap in the understanding of Hb biology in the placental mammal superorder of Laurasiatheria.

From its emergence, plants and animals adapted the porphyrin ring for diverse functions in both chlorophyll and heme proteins (e.g., O_2_ transport). That evolution, which led to the creation and expansion of Hb genes, continues to be pronounced to this day. Based on homology between invertebrates and vertebrates, the ancestral heme-containing globin gene present ~800 million years ago (MYA) appears to be Neuroglobin [12]. Since that time, gene duplications have resulted in a total of five gene families in tetrapods (amphibians, reptiles, birds, and mammals): neuroglobin, α-globin, β-globin, myoglobin, and cytoglobin. Evolutionary adaptation of Hb genes is recognized in fish, amphibians, reptiles, birds and mammals [9]. Additional understanding of Hb protein function, gene regulation, and evolution comes from studies of diverse species in which known environmentally-induced adaptations occurred [12], including extinct species such as the woolly mammoth [13].

The α-globin genes of amniotes are ζ-, μ-, and α- globin, plus θ-globin in marsupials/placental mammals [12]. ζ-globin is expressed in embryonic erythroid cells and α-globin is expressed in fetal and adult erythroid cells. μ- and θ- globin are transcribed in tetrapods, but their protein products have not been detected in mammals (whereas birds express μ-globin protein in adult erythroid cells). Placental mammals (eutherians) exhibit a relatively stable complement of α-globins, but a high level of diversity in their repertoire of β-globin genes [12]. β-globin genes were present in early vertebrates and their numbers were expanded through duplications within separate lineages. The stem eutherian contained five β-globin genes in one cluster, in the order 5′-ε-γ-η-δ-β-3′; these were derived by duplications of a single ancestral embryonic-like globin (resulting in ε, γ, η) and one adult-like globin (resulting in δ, β). Some β-globin genes were lost or duplicated in different species (some are extant as pseudogenes). The obscure η-globin gene is only extant in Laurasiatheria and its expression is only known for goat (embryonic [14]); however, the gene was lost in rodents and rabbits, and is a pseudogene in primates. γ-globin has the opposite pattern, where it is extant in Euarchontoglires (primates, rodents and rabbits), but absent or a pseudogene in Laurasiatheria.

Laurasiatheria is a very large and diverse suborder that together with Euarchontoglires makes up Boreoeutheria (the two other superorders of eutherians are Xenartha and Afrotheria) [15]. The two suborders are closely related according to genetic sequences, and it is believed they split ~85 MYA, after the break-up of the supercontinent Pangea into Laurasia (which includes North America, Europe and Asia) and Gondwana (Antarctica, South America, Africa, Madagascar, Australia, the Arabian Peninsula and the Indian subcontinent). In addition to carnivores such as dogs, cats and bears, Laurasiatheria includes shrews, hedgehogs, most hoofed animals (including horses, pigs, ruminants, camels, rhinos and hippos), pangolins, whales, and bats. Laurasiatherians are thus in an excellent position to shed light on the ancestral functions of Boreoeutherian β-globin genes, such as η-globin discussed above. Another mystery that they may help resolve is the significance of δ-globin. In humans, the δ-globin protein sequence has diverged significantly more than β-globin from the common δ/β-ancestor, and it is expressed at very low levels as a subunit of the minor adult Hb (HbA_2_, 3% of total adult Hb) [16]. Human δ-globin is thought to be physiologically irrelevant because it shows no clinical manifestations when mutant (and, despite having similar function to HbA, HbA_2_ levels are too low to replace HbA function in β-thalassemia major) [16, 17]. However, the δ-globin gene *HBD* has reduced diversity levels in humans, and it and the proximal pseudogene *HBBP1* have the strongest signatures of purifying selection at the β-globin locus [18, 19]. The facts discussed above have led Moleirinho et al. to propose that the evolutionary selection at *HBD* has to do with conservation of regulatory functions on other β-globin genes rather than δ-globin protein function [18].

Due to the high prevalence of hemoglobinopathies in people, α- and β- globin gene clusters of humans, and of the animal models, mouse and chicken are well characterized [10, 16, 20]. Despite the increasing importance of dog models of human diseases [21], almost nothing is known about canine Hb [22–25]. The reference annotation of dog Hb gene expression is limited to amino acid sequencing of isolated protein in adults. Those reports from 1969 and 1970 referred to simply α- or β- globins (without distinction between HBB and HBD) and concluded dogs only have one β- and two α- globin genes and that dogs lack fetal Hb [22–24]. As far as we are aware, there have been no updates of those studies. Using subsequent phylogenetic studies of α- and β- globins, one could begin to understand the gene complement of both. However, none of those studies focused on dogs, and their findings are not completely consistent – for example, in 2008, Opazo et al. showed the existence of the same set of β-globin genes we report here, but in 2012, Hardison showed the existence of all of those except *HBD1,* and Song et al. reported the existence of two *HBB* and one *HBD* genes in dogs [12, 26, 27]. Both of those latter studies, as well as those of Song et al. and Gaudry et al., included figures showing the chimeric *HBB/HBD* (our *HBD2* gene) gene in dogs that we report here [28, 29]. However, Song et al. suggested a different chimeric gene, *HBD/HBB* (our *HBB* gene). Because dogs were not the focus of any of those evolutionary studies, there was little, if any, elaboration or discussion of the data on dogs. Here we report the comparative genomics of the canine hemoglobin genes, which have important biomedical relevance.

## Results

### Comparative genomics of the canine α- and β- globin gene-cluster loci

**Fig. 1:**
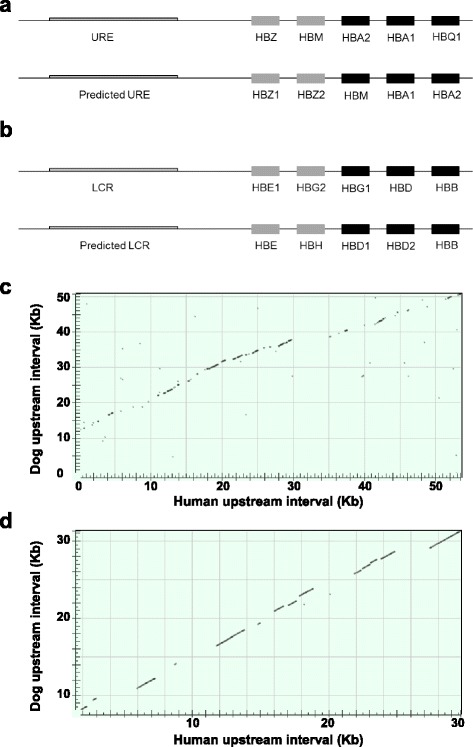
Genome structure of canine α and β globin gene clusters. Diagrams showing the arrangement and nomenclature of the α globin (**a**) and β globin (**b**) genes in humans (*above*) and dogs (*below*). Alignment of human and dog α globin (**c**) and β globin (**d**) upstream regulatory sequences (*HBE1* and *HBE* genes are shaded, for reference)

Using the relevant proteins and genes from humans and several other mammals to computationally align with the dog genome (BLAST/BLAT algorithms; canFam3.1 assembly), the canine α and β globin gene clusters were identified in chromosomes 6 and 21, respectively. Five genes constitute each one of the clusters, and all of them have the same basic globin structure: 3 exons and 2 introns), and are arranged in developmental order. The α-globin gene cluster is formed by three embryonic-like (*HBZ1*, *HBZ2* and *HBM*) and two adult-like (*HBA1* and *HBA2*) genes (Fig. 1a, Additional file 1). There are two duplicated genes in this cluster (same coding/protein sequence, but different intronic sequence): *HBZ1* and *HBZ2* (which have identical protein sequence), and *HBA1* and *HBA2* (same protein sequence except for one amino acid change [Ala/Thr] in position 131). The β-globin cluster has five β-globin genes: two embryonic/fetal-like genes (*HBE* and *HBH*) and three adult-like genes (*HBD1, HBD2* and *HBB*) (Fig. 1b, Additional file 1). As in the α-globin cluster, β-globin genes are arranged in developmental order, and there is a partially duplicated gene in this cluster: *HBD1* and *HBD2*, which have the same protein sequence, but different intronic sequences. Gene names used here are consistent with previous phylogenetic analyses of the globin genes in different mammals by Opazo and others [27]*.*

The expression of α- and β- globin genes is regulated by upstream regulatory regions called αURE and βLCR, respectively. Given the homology found between humans and dogs in the α- and β-globin gene clusters, we further analyzed upstream regions in order to investigate similarities between dog and human regulatory elements. Based on regulatory region extension reported in the human literature, we selected 60 Kb upstream of the first embryonic α-globin gene in the human and canine sequence (*HBZ* and *HBZ1*, respectively) and aligned them in order to assess the similarities and sequence conservation between αUREs (Fig. 1c). We repeated the same analysis for the βLCR, selecting 30 Kb upstream of the first embryonic β-globin human and canine gene (*HBE*) and aligned the sequences (Fig. 1d). Results demonstrate that the canine and human genomic sequences upstream of α- and β-globin clusters are well conserved, suggesting a similar regulatory role of upstream sequences.

### Evidence of “evolutionary” selection under domestication at the beta globin locus

**Fig. 2:**
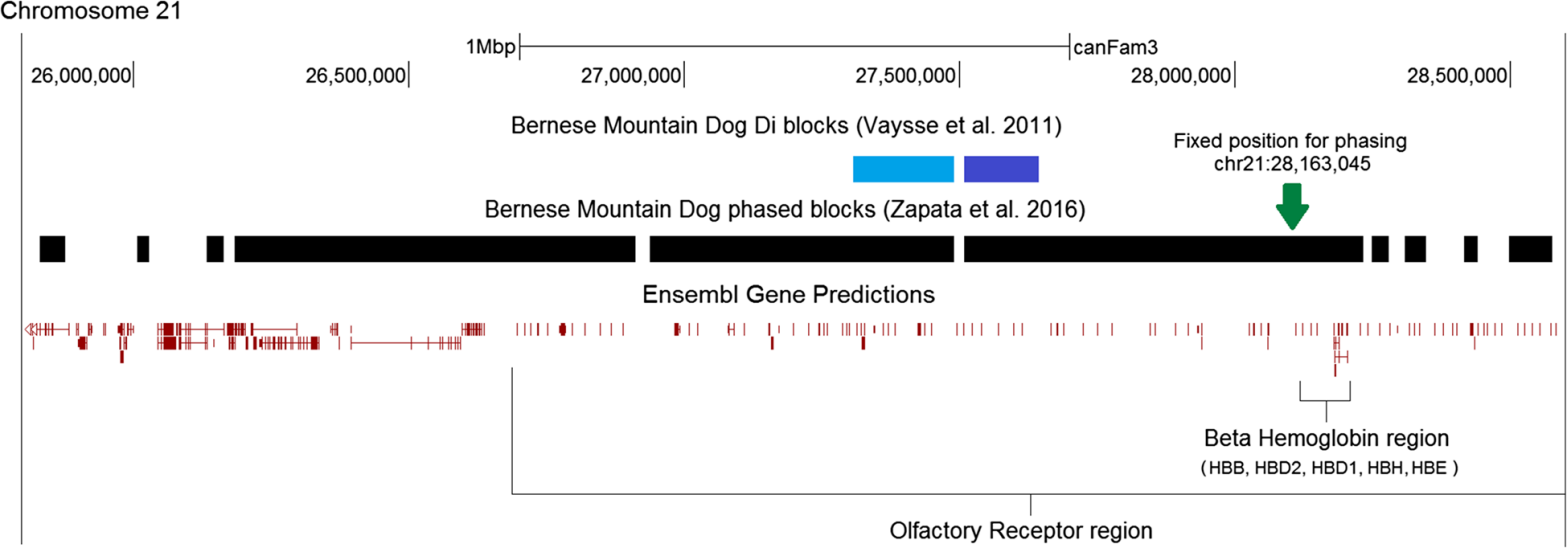
Evidence of evolutionary selection spanning the beta globin locus in the Bernese Mountain Dog. There are two types of evidence that indicate that there has been “evolutionary” selection under domestication in this locus. The *D*_*i*_ statistic for population differentiation shows the strongest signal in the genome of the Bernese Mountain Dog is in this region (marked in *dark blue*, with a second *D*_*i*_ segment abutting it shown in *light blue*) [4]. We used that same data of Vaysse et al. to conduct direct phasing of haplotypes anchored in the SNPs nearest the beta globin locus (*black bars* show phased haplotype segments; *arrow* shows one of two nearby SNPs that show the same haplotype pattern). No other breed showed a large phased haplotype at this locus. One phased haplotype block overlaps the top *D*_*i*_ region and contains the full beta globin locus (transcribed right to left here). Almost all the other genes in that segment appear as single ticks because they are intronless olfactory receptor genes

It was recently reported that indigenous Chinese dogs, including breeds such as the Tibetan Mastiff, carry coding variation in *HBB* as well as in *EPAS1* (which encodes HIF-2alpha, a transcription factor that regulates levels of red blood cells according to oxygen levels; this gene was previously shown to be under evolutionary selection for adaptation to high-altitude in humans [30–32]) [33]. Notably, that variation is strongly implicated to be under selection because their allele frequencies are directly correlated with altitude [34]. We thus evaluated Vaysse et al.’s previously published dataset of genotypes and statistical analysis of evolutionary selection from 509 dogs belonging to 46 breeds [4]. The Bernese Mountain Dog has its peak signal for genomewide population differentiation at the ß-globin locus (*D*_*i*_ statistic, *P* = 0.001, FDR = 0.13) (Fig. 2). We conducted direct haplotype phasing for the single nucleotide polymorphisms (SNP’s) in the same dataset, anchoring haplotypes with the SNP’s within or closest to the ß-globin locus (see Methods). This revealed that, of the dozens of diverse breeds in Vaysse et al. only the Bernese Mountain Dog has a fixed, very large haplotype block indicative of evolutionary selection (~750 kb, with strong signal of a much larger ancestral haplotype; the next largest is 155 kb and the median for all other breeds is ~40 kb). Importantly, one haplotype block overlaps both the peak *D*_*i*_ region mentioned above and the full ß-globin gene cluster. Most of the other genes in this haplotype block are olfactory receptor genes. In Additional file 2, we show that three breeds have potential signal for evolutionary selection overlapping the *EPAS1* gene – most significantly, the existence of a common ~3.1 Mb phased haplotype in the Doberman Pinscher. Because other genome regions in the genotype data for that breed have similar haplotype sizes to those of other breeds, this is not due to close-relatedness.

### Gene expression analysis of α- and β- globin genes in dogs and other carnivores

**Fig. 3:**
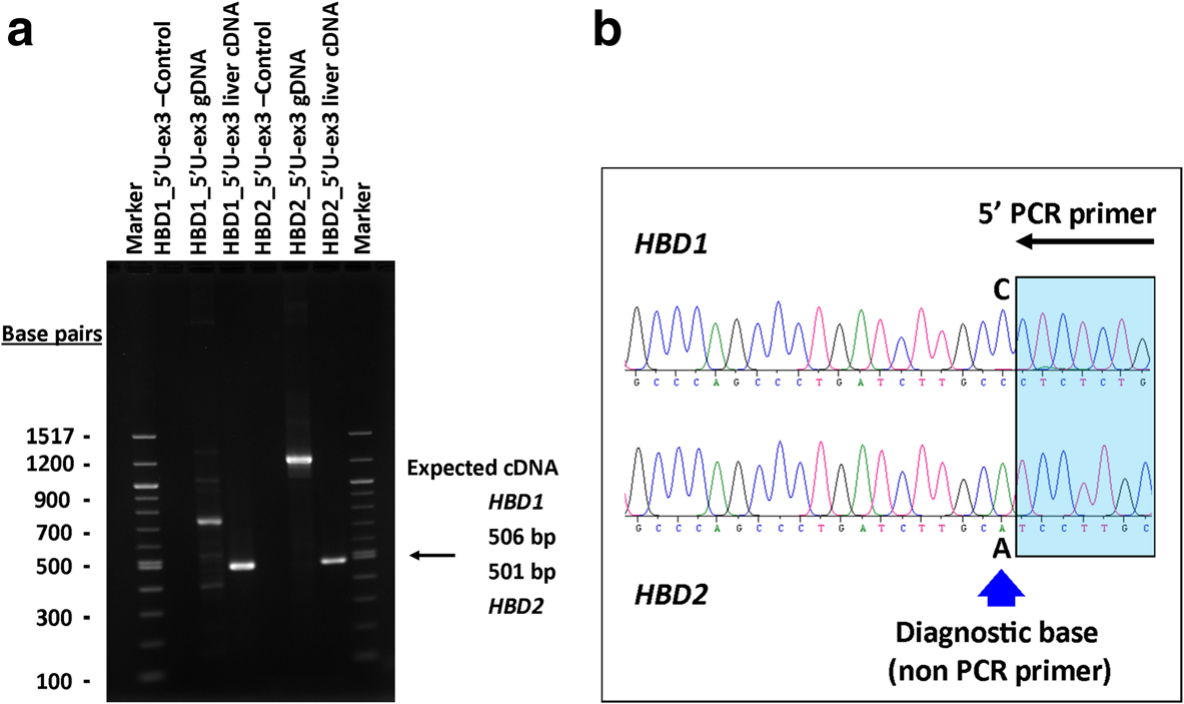
Expression of *HBD1* and *HBD2* mRNA in adults. **a** Primers specific for *HBD1* and *HBD2* 5′-untranslated regions were used with a primer to shared sequence in exon 3 to conduct PCR amplification of adult liver cDNA (along with controls of genomic DNA and no DNA template). **b** Standard dideoxynucleotide sequencing was used to test for the single non-primer position that differs between the two amplicons. Sequencing with the exon 3 primer showed the *HBD1* and *HBD2* cDNA amplicons are pure and unique (i.e., the respective diagnostic base is present in each), showing the two genes are expressed

We tested which computationally-predicted α- and β- globin genes from dogs are expressed in adult blood and liver cDNA. Each gene was queried by PCR amplification and sequencing. Our findings demonstrate that all ten of the predicted canine α- and β-globin genes are expressed and spliced in adults. The exons of *HBD1* and *HBD2* are identical and their transcripts were amplified using unique 5′-untranslated sequence primers and confirmed through sequencing of a non-primer single-base difference in the 5′-untranslated region. We thus confirmed that both canine *HBD* genes are expressed (Fig. 3), but have not yet determined their relative expression levels. [We have been unsuccessful in our attempts to amplify pre-mRNA cDNA using one pair of exon primers – i.e., shared identically in both genes – in order to use unique intervening intron sequences to measure their ratio.]

We also performed computational analysis of adult β-like globin gene expression. mRNA levels were measured through expressed sequence tags (ESTs) and this showed that the identical *HBD1/2* spliced-mRNA sequence is abundantly expressed in dogs whereas *HBB* is undetectable. Specifically, stringent BLAST analysis of the Genbank EST database using spliced segments of *HBD* yielded 422 hits for the full spliced-product of exons 1 and 2, and 1,038 hits for that of exons 2 and 3 (100% coverage and 100% identical for both). In contrast, there were no high stringency hits for the same analysis of *HBB*. The primacy of *HBD* is consistent with the original amino acid sequence from purified adult dog Hb [23], and by our prior isolation of adult dog blood Hb for crystal structure studies; the β-globin protein sequence of both of those studies corresponds to the δ-globin chain encoded by *HBD1/2* [35]. This finding is the opposite of humans and other members of Euarchontoglires, where *HBB* is the predominantly-expressed adult β-globin (accounting for 97% of the adult β-globin chains), and *HBD* has acquired many variations, is weakly expressed and has unknown significance.

To determine whether the adult-primacy of *HBD* expression is a unique feature of dogs, we studied the adult β-globin genes from another carnivore – the domestic cat (*felis catus*; NCBI GenBank). We found that the reference cat *HBB* protein (HBB_FELCA, NCBI accession P07412) [36] is incorrectly given as the actual sequence of cat *HBD*. Using both the cat genome assembly and, to resolve a gap, NCBI High Throughput Genome Sequence data, we determined the gene and protein sequence of cat *HBB* for the first time (Additional file 3). We thus established that cats have one gene each of *HBB, HBD, HBE* and *HBH* (Additional file 3). Notably, the prior work on cat *HBD* isolated from blood (presumed at the time to be *HBB*) established that *HBD* is the primary adult β-globin subunit in cats (accounting for 60–70%, with the remainder being *HBB*) [36]. By comparing to dog and cat *HBB* and *HBD*, we determined that the reference *HBB* protein isolated from ferret (another member of carnivora) adult-blood also appears to be incorrectly given as that of *HBD* (*Mustela putorius furo*; HBB_MUSPF, NCBI accession P68044) [37]. Ferret *HBD* was reported to be the only detectable adult β-globin [38]. The other carnivore for which there is expression data, the walrus, also expresses 100% *HBD* [39]. Lastly, we found 11 adult beta globin ESTs expressed in adult heart and liver of the American black bear (*Ursus americanus*) (accession numbers given in Methods). All of those were approximately full length cDNAs and appear to be the same gene sequence except for two variable positions that distinguish 7 vs. 4 of the sequences (neither of those is a coding variant). Comparison of that gene at the nucleotide and protein levels to two other carnivores for which the genome structure of this locus is available (dog and cat) strongly suggests the gene is *HBD*. Thus all 11 ESTs expressed in adult bear appear to be *HBD* and none *HBB*. This is consistent with carnivores predominantly expressing *HBD* as the adult beta globin. These findings suggest that the biology of adult β-globins in dogs – the primacy in adult expression and the amino acid conservation of *HBD* – may be a general property of carnivores. Based on the other branches of Laurasiatheria for which data are available, the primacy of *HBD* in adults may be unique to carnivora. Specifically, the following all express *HBB* as the major β-globin in adults: black flying fox, horse, white rhinoceros, camel, alpaca, and pig [28, 40–43]. Outside of carnivora, we found no evidence that other members of Laurasiatheria express *HBD* as the primary adult globin. We thus find that the primary adult beta globin for five of five carnivores is *HBD* and for six of six non-carnivore members of Laurasiatheria it is *HBB*.

### Analysis of chimerism in canine β-globin genes

The amino acid sequences of mammalian *HBB* and *HBD* indicate that they arose by duplication of one those genes. The arrangement and structure of the canine globin gene clusters is similar to their human counterpart. Both human and canine lineages evolved from a common ancestor that had both β- (*HBB*) and δ- (delta, *HBD*) genes in the β-globin gene cluster. Human *HBB* is ancestral-like in protein sequence and highly expressed (major adult Hb) and *HBD* is highly divergent and minutely expressed (minor adult Hb). However, we found that in dogs it is the opposite: *HBD* – and not *HBB* – is the evolutionarily conserved and highly expressed β-globin (see next paragraph; [27]). We know the dog genes are indeed the orthologs of *HBB* and *HBD* (vs. having swapped positions in the carnivore lineage) because the flanking and intronic sequences are sufficiently conserved to establish that (but see chimeric gene findings below; and [27]).

**Fig. 4:**
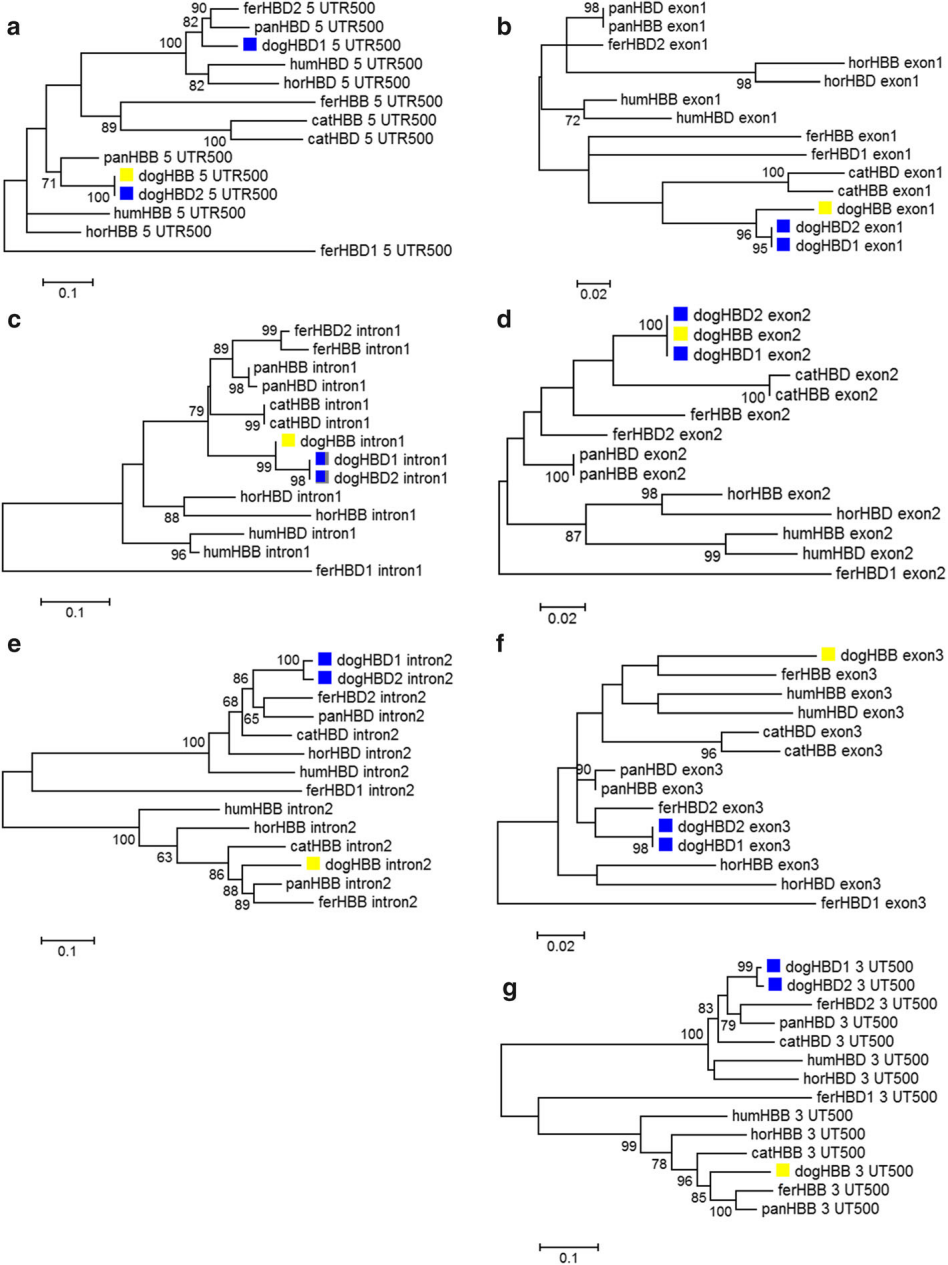
Phylogenetic analysis of canine *HBD* and *HBB* genes shows gene chimerism between them. Maximum Likelihood trees were generated for gene segments from carnivores, with human and horse as an outgroup. **a**-**g** correspond to gene structure elements: 5′UTR (316 nucleotide positions were analyzed after complete elimination of gaps), exon 1 (92 positions), intron 1 (83 positions), exon 2 (223 positions), intron 2 (420 positions), exon 3 (125 positions) and 3′UTR (351 positions), respectively. Dog *HBD1* and *HBD2* genes are marked blue and dog *HBB* yellow. Abbreviations: 5′/3′ UTR500, first 500 bases of 5′ and 3′ untranslated region sequence; fer, ferret; pan, panda; hum, human. Tree branch lengths correspond to the number of changes per amino acid position (scale bar). Bootstrap values were generated from 500 repetitions. The same topology of the dog genes is evident using the Maximum Parsimony treeing method (Additional file 7)

As noted in the background section, there have been conflicting reports regarding the description of chimeric β-globin genes that have resulted from gene conversion. In Fig. 4, we show phylogenetic analysis of canine *HBB*, *HBD1* and *HBD2* evaluated together with those from other carnivores – cat, ferret and panda – as well as human and horse for comparison. [Inconsistent reports show a chimeric *HBB/HBD* (*HBD* gene with undetermined *HBB* sequence in the 5′ half) in human [12] (that was not shown in [28]); but both reported the horse as having one true *HBD* and one true *HBB* (i.e., no chimerism).] We found that dog *HBD2* acquired the promoter region of *HBB* through a gene conversion event (consistent with [27, 28]). We also found that *HBD1*, *HBD2* and *HBB* all share an identical exon 2 as the result of gene conversion(s) (as well as 6 and 8 bases of the abutting introns 1 and 2, respectively). Notably, many of the comparisons of gene segments show greater similarity across paralogs within species, than vice versa (i.e., they appear to be monophyletic). It is thus difficult to determine the origins of segments such as the dog exon 2 shared by *HBD1*, *HBD2* and *HBB*.

**Fig. 5:**
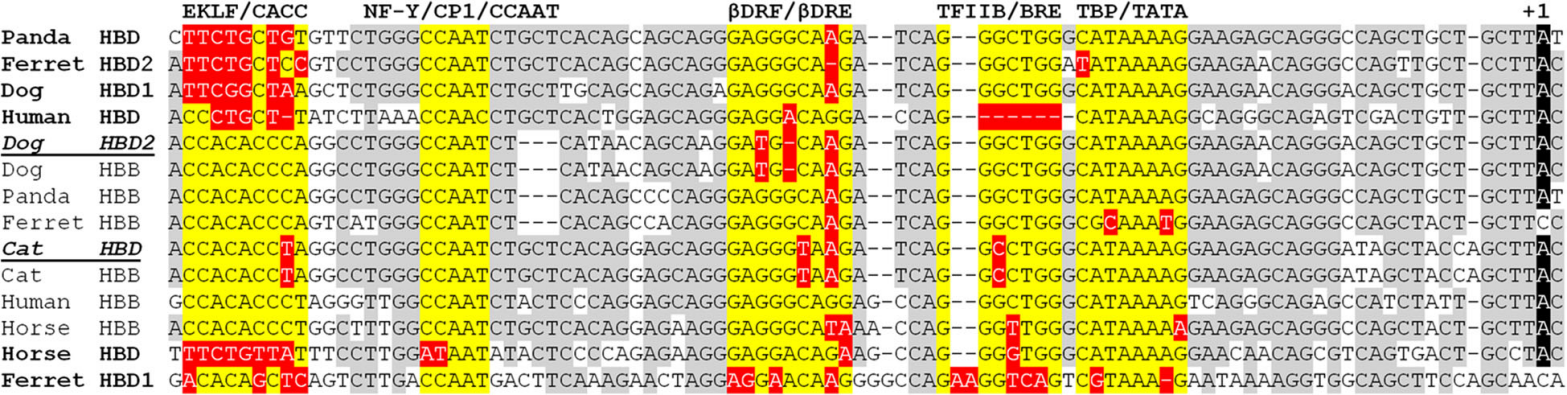
Promoter analysis of canine adult β globins. Canine *HBD1*, *HBD2* and *HBB* are aligned with their carnivore (panda, ferret and cat) and human and horse orthologs. Sequences known to be bound by regulatory proteins are shown in yellow if they match the consensus and red if they are non-consensus. Dog *HBD1* appears to carry the ancestral *HBD* promoter sequence, whereas both dog *HBD2* and cat *HBD* (italics/underline) show evidence of gene conversion with acquisition of the full *HBB* promoter sequence. As a result, those genes now have consensus CACC boxes, which are known to be bound by erythroid Krüppel-like Factor (EKLF) to activate *HBB* expression

In Fig. 5 we show a multiple sequence alignment and promoter analysis. This clearly shows that a canine *HBD2* gene conversion event at its 5′ end has resulted in an *HBB*-identical proximal promoter. Similar to previous reports of 5′ chimerism in the cat *HBD* gene [27, 28], that includes the proximal promoter from *HBB*. Thus both cat *HBD* and dog *HBD2* have acquired the full complement of evolutionarily-conserved regulatory sites otherwise present in the *HBB* but not *HBD*. In contrast, human, horse, panda and ferret have distinct *HBD*- and *HBB*-like promoters with the former lacking key regulatory DNA-binding sequences. Curiously, *HBB* from dog, ferret and panda have a 3-bp deletion relative to cat (and human/horse); one possible explanation is that the ancestral carnivore *HBB* acquired this deletion but it was replaced through gene conversion in the cat lineage. Analysis of human variants near the *HBB* conserved DNA-binding sites revealed one conserved position that is divergent in dog *HBB/HBD2* (G > A, two bp upstream of the βDRE sequence) and has been reported as variant in the human HbVar database (but is not known to be disease associated; [44]).

### Evolutionary analysis of adult β-globin proteins of dogs

We next compared the amino acid sequence of canine β-globins to that of diverse mammals in order to determine if they are well conserved. We used the TreeFam database of proteins derived from genome sequences of model organisms selected for representation of extensive phylogenetic diversity. We collected all mammalian β-globin proteins annotated in TreeFam and removed those with sequence gaps or other features that suggested likely sequencing or assembly errors (i.e., significant insertions or deletions). Only one species was kept for closely related pairs, specifically mouse/rat and human/chimpanzee. We then conducted phylogenetic treeing of all remaining β-globin sequences and removed a small number of sequences that did not branch clearly (with high bootstrap confidence values) with only adult-like or embryonic-like β-globins. We conducted multiple sequence alignments of the final sets of adult (n = 35) and embryonic (n = 43) β-globins, and generated a Sequence Logo display of the frequency of any amino acid at each position across mammalian phylogeny (Additional files 4 and 5).

**Fig. 6:**
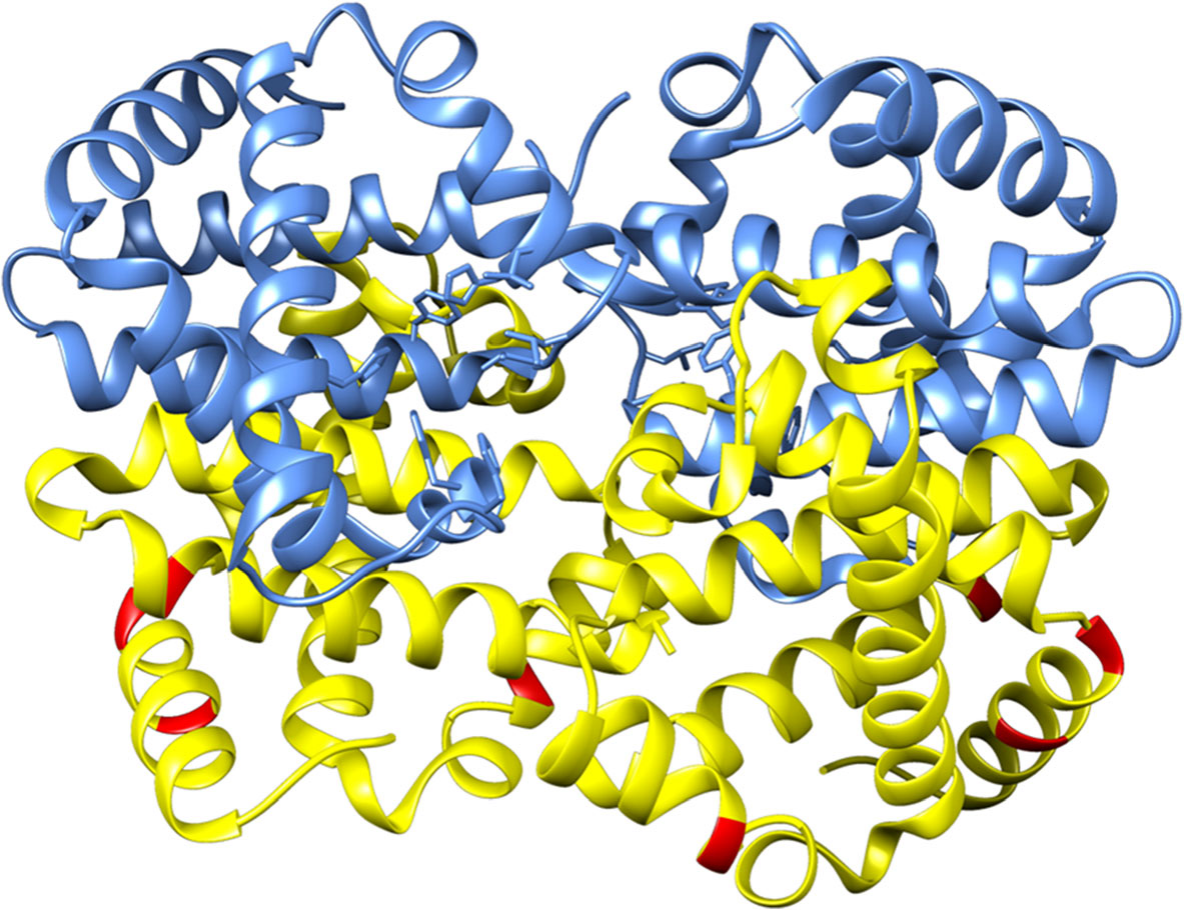
Predicted 3D structure of dog HBA-HBH tetramer. α chains are shown in blue, and γ chains in *yellow*. Notable amino acid positions in HBH are highlighted *red*. Those positions are: I at that position 13 (canine HBH is the only one of the 43 to have it); R at position 18 (HBH is one of only two having it, since 38 are K and 3 are Q at this position); 88E, which is unique in dog HBH (although 2 others are D); and HBH 127 T is unique (the others are only V/M/L/I)

Dog β-globin proteins cleanly branch with embryonic (HBE and HBH) or adult (HBB and HBD1/2) β-globins, and are the same length and lack large amino acid sequence changes compared to the other members of the family. However, canine β-globins have several notable amino acid positions. For embryonic β-globins, that includes i) canine HBH is the only one of the 43 to have I at position 13; ii) HBH is one of only two with R at position 18 (38 are K and 3 are Q); iii) HBH 88E is unique (although 2 others are D); and iv) HBH 127 T is unique (the others are only V/M/L/I) (Fig. 6). In contrast, HBE lacks any such rare substitutions. Further studies are necessary to compare canine HBH to all available HBH sequences to determine whether the dog protein is uncommonly divergent. A curious feature of the amino acid frequency analysis across adult β-globins from diverse mammals is that no position has I as the most common amino acid. However, there are 18 such positions each for L and V (and 1 M in addition to the initiation codon). Presumably this is not due to the general incompatibility of I in alpha helices, as V also has that feature. For adult β-globins, the following canine positions are potentially interesting: i) HBB/HBD1/2 L11 is unique among the 35 adult β-globins (although some of the most divergent proteins here, from shrew and bat, have V and I, respectively); ii) HBB M15 is unique (the others are all L, except for a highly divergent Guinea pig protein which is V); and iii) HBB R121 is unique (the others are all K, except for proteins from pig and Guinea pig: H and S, respectively). Further structure-predictions and functional studies will be necessary to determine the significance of these amino acid variants.

## Discussion

While the reductionist approach to biology has been a success, the next phase is to understand biology at the organismal and ecological levels [45]. We propose that the dog is an ideal translational genomic model to accelerate discovery and development of therapies [1]. Hemoglobin biology was the earliest and arguably the most highly developed topic of investigation in the molecular biology era. It is studied at the level of human disease (thalassemias and sickle cell), comparative genomics, evolutionary adaptation, developmental gene regulation, epigenetics and chromatin structure, post-translational regulation, protein structure, and protein turnover. However, the field has not yet met the highest goals of those efforts [1]. Those aims include efficacious and widely-available methods to generate blood substitutes or to address thalassemias/sickling (e.g., by re-activation of fetal β-globin expression or genome editing and transplantation [46]). Inbred mouse models of human hemoglobin have been invaluable, both in experimental and evolutionary studies. However, one of their greatest biological advantages – genetic simplicity – is also a liability. That is because it is not clear what all of the organismal effects are of strain-selection for fecundity, and, related to that, it is generally unknown how representative any mouse strain biology is of wild mice (or humans). Understanding human biology has the opposite problem: people have high levels of variation and heterogeneity of complex traits, making it difficult to understand gene-environment-phenotype interactions.

Dogs add unique and powerful dimensions to biomedical research [2, 21]. In the USA alone there are ~75 million dogs, a major proportion of which live in human environments and receive a high level of health care. The evolutionary and domestication history of dogs have resulted in hundreds of breeds, each with vastly reduced genetic variation and trait-heterogeneity. And breeds can be classified into approximately ten groups defined by genetic relatedness. These factors make dogs ideal for genetic mapping of complex traits and for understanding gene-gene and gene-environment interactions. Because of recent developments in sequencing, it will be simple to identify breed variation in the α- and β- globin loci (or in other loci associated with hematological traits [8]). Many breeds are indicated for this analysis due to biological relevance of selection-traits – such as racing Greyhounds and sled-racing Siberian Huskys. Others have reported high altitude-acclimated breeds like the Tibetan mastiff have evolutionarily-selected for β-globin variation. In the present study, we show evidence that a haplotype containing the β-globin locus has the strongest signal of evolutionary selection in the genome of the Bernese Mountain Dog which originated in Dürrbach, Switzerland (average elevation 466 m), within the canton of Berne (elevation range 402–4,274 m). Once such variants are identified, breeds segregating those can be studied to compare wild type and variant homozygotes. Such findings could thus be rapidly dissected in clinical dog studies or in induced pluripotent cells; and those could be validated in mouse model and biophysical studies. Genome editing would then allow precise therapies to be created and tested, including in clinical trials in pet dogs with severe disease. Our rich hemoglobin gene findings at such a late stage of the genomics era show that, despite the tremendous accomplishments of the field of dog models, genome annotation is in its infancy.

In humans and other members of Euarchontoglires for which it is known, the β-globin chain (*HBB*) is the predominantly-expressed β-globin in adults. The other adult-like β-globin chain δ (*HBD*) in those species is thought to be dispensable at the protein level, and it has been proposed that signals of evolutionary selection within human *HBD* are due to roles in the regulation of other genes at this locus [18]. However, there is a high frequency of inactivated, deleted, duplicated and chimeric *HBD* and *HBB* genes across mammals [27, 28]. For example, rats have four *HBB* genes and a single inactive *HBD* (pseudogene), whereas European hedgehogs have three *HBD* genes (each has *HBB* sequence in its 5′ end), one *HBD* pseudogene and no *HBB* gene. Thus, the overall pattern does not suggest that either δ- or β- is critical or dispensable, but rather that at least one adult type β-globin gene is necessary and multiple copies of either of them may be evolutionarily advantageous.

Here we show that the δ-chain is the primary β-globin in adult dogs and other carnivores, a property that appears to not apply more broadly to other branches of Laurasiatheria [28]. Our promoter analysis of adult globins from dog and cat indicates possible mechanisms by which *HBD* may be expressed at higher levels than *HBB*: i) in both species, the *HBB* promoter region with the full complement of regulatory sites necessary for normal adult expression have replaced the *HBD* promoter region (in dogs, only for *HBD2*); and ii) *HBD* is upstream of *HBB* and could be preferentially expressed even if the two genes had identical DNA sequences. Cat adult β- globin expression is consistent with that, the single *HBD* and *HBB* genes have identical *HBB* promoters (through gene conversion), and the expression levels in adults are approximately 65 and 35%, respectively [36]. In the case of dogs, the predominance of *HBD* expression is far greater than it is in cats; it seems likely that this is due to additional DNA sequence differences between the species. Dogs have two *HBD* genes, the first with an *HBD* promoter and the second with a recombined *HBB* promoter. Conventional understanding would suggest that *HBD1* does not express strongly in adults because it lacks the critical EKLF/CACC motif that is present in *HBB* and acquired by *HBD2* [10]. However, we showed here that both *HBD1* and *HBD2* are expressed. Because the two genes have identical exon sequences and we were not successful in our attempts to PCR-amplify cDNA from pre-mRNA (i.e., to use unspliced-intron sequence to distinguish between the two genes amplified with common primers), we have not yet determined their relative levels; we plan to do this in future studies.

Another evolutionary question that our findings begin to address is the significance of the *HBH* gene. The common ancestor of the two major clades of placental mammals had the full complement of embryonic β-globins: *HBE*, *HBG* and *HBH*. However, extant placental mammals have one or two copies of *HBE* and either *HBG* or *HBH* – but never both (very rarely, both of the latter two genes may be absent). Dogs have one *HBE* and one *HBH* gene. The protein sequence of dog HBE is evolutionarily highly-conserved, but that of HBH has three positions with very rare substitutions. Similarly, the dog HBD1/2 sequence is evolutionarily conserved, but HBB has multiple rare substitutions. A curious observation about adult β-globin proteins across mammals is that they have many positions in which leucine (n = 18 AA positions) and valine (n = 18) are the most common amino acid, but none in which isoleucine is the most common.

## Conclusion

We have determined the comparative genomics of dog hemoglobin genes. This establishes several important questions that are likely to lead to important new understandings of hemoglobin biochemistry, genetics and evolution. With respect to biochemistry, it will be interesting to figure out the effects of different subunit compositions and atypical amino acid substitutions on hemoglobin structure and function. Among the questions pertaining to genetics, the chimerism and regulation of gene expression of β-globins *HBB* and *HBD1/2* are likely provide new insights. Lastly, our study has highlighted several evolutionary questions, including the biological significance of the *HBH* and *HBD* subunits which are currently rather mysterious. A particularly intriguing issue is how and why carnivores predominantly express *HBD* and not *HBB* in adults. We speculate that the very high level of chimerism and the fact that there are approximately 400 extant dog breeds signify that it is not improbable that chimeric polymorphisms under selection will be discovered. Similarly, it will be interesting to identify coding and regulatory variation in hemoglobin genes, and then to determine their effects on physiology, environmental adaptation and disease.

## Methods

### Computational analysis

We used the Basic Local Alignment Search Tool (BLAST) the BLAST-like alignment tool, and NCBI and UCSC Genome [47–50] browsers to map the dog and cat hemoglobin genes querying with human and other mammalian genes and proteins. Protein alignments were done using ClustalW2 [51] and consensus sequences were obtained using Weblogo [52]. Molecular structure was predicted using Phyre2 [53] and the 3D structure was created using UCSF Chimera software [54]. American black bear *HBD* EST accession numbers follow: GW280247, GW278169, GW285405, GW283884, GW295575, GW278208, GW281322, GW279806, GW290503, GW290811 and GW284694.

### Direct haplotype phasing analysis

Phasing was done as described by Zapata et al. [7]. To construct the phased haplotypes we selected the closest SNP marker to the locus of interest and separated their carrier status as heterozygous or homozygous. Since direct phasing can only be done on the homozygous, all heterozygous individuals were excluded. Homozygotes were divided for analysis of all A and B allele dogs separately. To construct the largest common phased haplotype we evaluated each marker upstream and downstream starting from the selected SNP and only kept SNP’s that had an allele frequency of at least 0.95 (this provides homozygous segmentation; visualization of the SNP pattern establishes the presence of a single vs. multiple haplotypes with the reference SNP). We only included breeds in our analysis that had at least 4 homozygous individuals per SNP variant. Allele frequency calculations were obtained using PLINK v.1.07.

### Phylogenetic analysis

Creation of a high-quality set of embryonic (HBE, HBG, HBH) and adult (HBB, HBD) β-globin proteins from placental mammals for comparative evaluation of dog β-globin variants: We used TreeFam (http://www.treefam.org/) to identify all annotated β-globin proteins from sequenced vertebrate genomes. Protein sequences were removed if they were duplicates, incomplete or appeared likely to have assembly/annotation errors. Separately from the TreeFam analysis, we manually curated a set of β-globin genes attempting to identify all for human and select species of macaque, galago, mouse, bat, dog, horse, shrew, armadillo and elephant. Together, this resulted in a set of 114 β-globin sequences. We aligned sequences using ClustalW (as implemented in the SDSC Biology WorkBench), and conducted phylogenetic treeing with bootstrapping using Mega 5.1 with default settings. Comparison of Neighbor-Joining and Maximum Parsimony methods yielded similar tree topologies that allowed for clean isolation of embryonic-like and adult-like β-globins from placental mammals. To reduce bias from closely related sequences, all rat and chimpanzee β-globins were removed. This resulted in sets of 43 embryonic-like and 35 adult-like β-globins (Additional files 4 and 5).

Phylogenetic analysis of *HBD* and *HBB* DNA sequences in dogs and select other species (Fig. 4; Additional files 6 and 7): Multiple sequence alignments were conducted with ClustalW2 [55]. Evolutionary analyses were conducted using the MEGA7 software package [56]. For Fig. 4, the Maximum Likelihood method based on the Tamura-Nei model was used to infer the evolutionary history [57]. The tree shown is the one with the highest log likelihood. Bootstrap values shown next to the branches give the percentage above 60 of trees in which the associated taxa clustered together. The initial trees for the heuristic search were automatically obtained using the Neighbor-Join and BioNJ algorithms to generate a matrix of pairwise distances estimated using the Maximum Composite Likelihood approach, and then choosing the topology with the strongest log likelihood value. The tree is drawn to scale, and branch lengths are given as the number of substitutions per site. All gap positions were deleted. The number of positions in the final dataset for each analysis is given in the figure legend. For Additional file 7, the Maximum Parsimony trees were generated with the Subtree-Pruning-Regrafting algorithm (pg. 126 in ref. [58]) with search level 1 (the initial trees were obtained by the random addition of sequences using 10 replicates).

### Globin gene expression methods

Blood RNA from two adult dogs was collected using the PAXgene blood RNA kit (QIAGEN Inc., Valencia, CA, USA), and cDNA synthesized using Superscript II Reverse Transcriptase (Life Technologies Corp., Grand Island, NY, USA). Liver RNA was collected from one adult dog using TRIzol reagent (Invitrogen, Carlsbad, CA, USA) according to the manufacturer’s instructions; and cDNA was generated using Superscript III First-Strand Synthesis System for RT-PCR (Invitrogen) according to manufacturer’s instructions. Exon-specific primers (Additional file 8) were designed to amplify the coding regions of all the different globin genes. Reverse transcriptase PCR was performed using JumpTaq polymerase (JumpStart REDTaq Hot Start DNA Polymerase, Sigma); Tm, annealing time and number of cycles was adjusted to individual primer sets in order to optimize amplification conditions. Products were purified (PCR purification/gel extraction kits; QIAGEN Inc., Valencia, CA, USA) and sequenced (Eurofins MWG Operon, Huntsville, AL, USA), and results obtained were analyzed using the computer software DNASTAR Lasergene Core Suite (DNASTAR Inc., Madison, WI, USA) and compared to the canine reference sequence (canfam3.1).

## Additional files

Additional file 1: Gene structure sequences of α- and β- embryonic and adult globin genes. (DOCX 125 kb)
Additional file 2:
*EPAS1* (HIF-2alpha protein) gene region may harbor variation under selection. Direct haplotype phasing anchored within (shown here by vertical line) or near the *EPAS1* gene show that three breeds have large (Irish Wolfhound and English Setter) or very large (Doberman Pinscher) phased haplotype blocks (shown as black horizontal bars). The latter two of those breeds also have evidence of population differentiation (*D*_*i*_ statistic, blue bars), and the Doberman Pinscher also has evidence of reduced heterogeneity (*S*_*i*_ statistic, red bars) [4]. (TIF 432 kb)
Additional file 3: Complement of β-globin proteins in the domestic cat. (DOCX 14 kb)
Additional file 4: A) Multiple sequence alignment of embryonic β-globins from model placental mammal genomes, and B) consensus sequence logo from those embryonic β-globins. (PDF 1094 kb)
Additional file 5: A) Sequence alignment of adult β-globins from model placental mammal genomes, and B) consensus sequence logo from those adult β-globins. (PDF 1065 kb)
Additional file 6: Taxonomy annotation of genes used for the phylogenetic analysis with identification number and species information. (XLSX 15 kb)
Additional file 7: Maximum Parsimony analysis of same data as in Fig. 4, phylogeny of *HBD* and *HBD* genes from dogs and select other species. (PNG 223 kb)
Additional file 8: Sequences of primers used for PCR and sequencing studies. (XLSX 45 kb)

## Abbreviations

βLCR: Beta locus control region
αURE: Alpha upstream regulatory element
cDNA: Complementary deoxyribonucleic acid
EST: Expressed sequence tag
Hb: Hemoglobin
HbA: Major adult hemoglobin (two α chains + two β chains)
HbA_2_: Minor adult hemoglobin (two α chains + two δ chains)
HBB: Hemoglobin beta subunit
HBD: Hemoglobin delta subunit
HbVar: Hemoglobin variants database
mRNA: Messenger ribonucleic acid
MYA: Million years ago
PCR: Polymerase chain reaction
SNP: Single nucleotide polymorphism
