# Supplementary material for: Comparative genomics of canine hemoglobin genes reveals primacy of beta subunit delta in adult carnivores
Source: BMC Genomics. 2017 Feb 8;18:141. doi: 10.1186/s12864-017-3513-0 (PMC5299747; doi:10.1186/s12864-017-3513-0)
Supplement: Additional file 1: — Gene structure sequences of α- and β- embryonic and adult globin genes. (DOCX 125 kb) [file 12864_2017_3513_MOESM1_ESM.docx]

>dogHBZ1_5’UT500

ccccagggatcatagcacctgagggaagggcctggctgcacgggctgcggggccccaaggagtgcggccagcagaacggcaggttccagcccaaggacagcgcgtggagggctaatcctctggcttcccccattctgtgactcccaatacaagagcgtggtcagagctgggctacacacacgggcatggcctcctgggctggctgggccctatctctccgggactctggtcaggctgtggccaacaccctctctaaggccccagatgggggtggggggtcttggtggagtgagagcccctcagcccagcgtcccaccaggccctgaccagccgtcctcactgggtctgataagacaccccacccccacggccctctcccctctccaggccagtggccacagcctggcccccaccctggctcctggtatataaagggatgctgggggctgagcaccccacaggccggccctgagcacacccagctcccactcagctccacc

>dogHBZ1_exon1

ATGTCTCTGACCAAGGCCGAGAGGACCATCATCCTGTCCATGTGGGGCAAGATCTCCACCCAGGCGGATGCTATTGGCACCGAGGCCCTGGAGAG

>dogHBZ1_intron1

gtcagtgcctgatgggctgcgctggggccaggaggtgacagtgagggttagtgaggcccggggtggggggcacagggtcagtgcgcaggaaggggaggtgcatggggaaggggtctggcacccaggtctcccccacctccacgcagtgagtgaaggaaggaaggaaggaactgctgagccatgggccgctggggtgggcttgtgccacgccccggggtgggggcccggggaccggggagagtcggccaggcgggcctgcccttgtgccacaccggggcgtgcgggtccacggggaggttcgcgtccctgtttctgtcctaaaacatccgttacaacgtgtcgtggggggaggacggggcttcccacgcgtcagtggcgccgccgtggcccgcag

>dogHBZ1_exon2

GCTCTTCGCCAGCTTCCCGCAGACCAAGACCTACTTCCCGCACTTCGAGCTGCGCGCGGGCTCGGCGCACCTGAGGGCGCACGGCGCCAAGGTGGTGGCCGCGCTGGGCGACGCGGTGCGCAGCCTCGACGACGTGGCGGGCGCCCTGTCCAGGCTGAGCGAGCTGCACGCCTACATCCTGCGCGTGGACCCGGTCAACTTCAAG

>dogHBZ1_intron2

gtgggcgggggggggggtccccagggtccccagggtccccaggcgcctcccgcgctcacccaggccctccctccggcgcag

>dogHBZ1_exon3

CTGCTGTCCCACTGTCTGCTGGTCACCCTGGCCTCGCACTTCCCCGCCGACCTCACGGCCGACGCCCACGCCGCGTGGGACAAGTTCCTGTCGCTCGTGTCCTGCGTCCTGACCGAGAAGTACCGCTGA

>dogHBZ1_3’UT500

gcgcccccagctgcgcccctgcgcagctcccgcacctcactgcgacaccccccctccccctcccacctcgctgacgcaccaataaaccagtgcacgtccacctcgcctccctggtcactggcaccgtggagggggctgcagggggtgggggcgacgggctgcaggggggtgggagctgcaggggaggggtggcgggcagcaggggtgtgggggctgcagcggagggggcggcgggctgcagggggggtgtggcaggctgcaggtggtggggagacgggctgcagagagggtcgatgggctgcaggcagcggcagactgctgggggagcgggggtggctgcaggtggggcgggctgcagggtggggctgctggaatcctccctcggaccccagagtctctgcgagggcagctggggatcccccggttgagcccccacccctcaggctgtccctggggcccccgtggcgctctctccttcctctggagtttgtactgcctcc

>dogHBZ2_5’UT500

aagactgtcatgctctgtgacagctggtctcagtctccgtctgtgaccctgtgttcactcatctgtaaagtgcttcctaaggtggccccatagagcagaagcagagggaggaggagagaatgcttctgtgagagcctgacacactccaggtcacccttccatcacaaggacagtgagcgtggaggccagtcacggggccgggctgacatggcgactctccacccgggcctgggcccctatctcccaggaacttggactgacctgtggcccagcagtggggaggcctgagtgagagcccctcagcccagcgtcccaccaggccctgaccagccgtcctcactgggtctgataagacaccccacccccacggccctctcccctctccaggccagtggccacagcctggcccccaccctggctcctggtatataaagggatgctgggggctgagcaccccacaggccggccctgagcacacccagctcccactcagctccacc

>dogHBZ2_exon1

ATGTCTCTGACCAAGGCCGAGAGGACCATCATCCTGTCCATGTGGGGCAAGATCTCCACCCAGGCGGATGCTATTGGCACCGAGGCCCTGGAGAG

>dogHBZ2_intron1

gtcagtgcctgatgggctgcgctggggccaggaggtgacagtgagggttagtgaggcccggggtggggggcacagggtcagtgtgcaggaaggggaggtgcatggggaaggggtctggcacccaggtctcccccacctccacgcagtgagtgaaggaaggaaggaaggaactgctgagccatgggccgctggggtgggcttgtgccacgccccggggtgggggcccggggaccggggagagtcggccaggcgggcctgcccttgtgccacaccggggcgtgcgggtccacggggaggttcgcgtccctgtttctgtcctaaaacatccgttacaacgtgtcgtggggggaggacggggcttcccacgcgtcagtggcgccgccgtggcccgcag

>dogHBZ2_exon2

GCTCTTCGCCAGCTTCCCGCAGACCAAGACCTACTTCCCGCACTTCGAGCTGCGCGCGGGCTCGGCGCACCTGAGGGCGCACGGCGCCAAGGTGGTGGCCGCGCTGGGCGACGCGGTGCGCAGCCTCGACGACGTGGCGGGCGCCCTGTCCAGGCTGAGCGAGCTGCACGCCTACATCCTGCGCGTGGACCCGGTCAACTTCAAG

>dogHBZ2_intron2

gtgggcgggggggggggtccccagggtccccagggtccccaggcgcctcccgcgctcacccaggccctccctccggcgcag

>dogHBZ2_exon3

CTGCTGTCCCACTGTCTGCTGGTCACCCTGGCCTCGCACTTCCCCGCCGACCTCACGGCCGACGCCCACGCCGCGTGGGACAAGTTCCTGTCGCTCGTGTCCTGCGTCCTGACCGAGAAGTACCGCTGA

>dogHBZ2_3’UT500

gcgcccccggctgcggccccgagcgccctcccctgcgtccctcccgcgtgtccccaataaaaggctgaggacggagcggctcccgcgttctgcatgttggcgggaagcaggggagggacgggggcgcgggggcgagaacctttgctccccgagtgtgcggccccccgcgcccccgcgccccggctctgcctgcggttccgcacccgctgcggcctgcgctgggccgccccccacggtgggccccgcagataagcgcgggcggcgcctgggcgcgggggggccgctataaggaggcgggcggggcgggagggcgcagagcagccccggcagcgccatgctcagcgcccaggagcgcgcccaggtagcgcaggtctgggacctgatcgcgggccacgaggcggcctttggggcggagctgctgctcaggtgggctgcggcgggggcgcgggccggggtgcctgggggcgctgggggcgggggccggggggtacccggggtgc

>dogHBM_5’UT500

gcctcccgcgctcacccaggccctccctccggcgcagctgctgtcccactgtctgctggtcaccctggcctcgcacttccccgccgacctcacggccgacgcccacgccgcgtgggacaagttcctgtcgctcgtgtcctgcgtcctgaccgagaagtaccgctgagcgcccccggctgcggccccgagcgccctcccctgcgtccctcccgcgtgtccccaataaaaggctgaggacggagcggctcccgcgttctgcatgttggcgggaagcaggggagggacgggggcgcgggggcgagaacctttgctccccgagtgtgcggccccccgcgcccccgcgccccggctctgcctgcggttccgcacccgctgcggcctgcgctgggccgccccccacggtgggccccgcagataagcgcgggcggcgcctgggcgcgggggggccgctataaggaggcgggcggggcgggagggcgcagagcagccccggcagcgcc

>dogHBM_exon1

ATGCTCAGCGCCCAGGAGCGCGCCCAGGTAGCGCAGGTCTGGGACCTGATCGCGGGCCACGAGGCGGCCTTTGGGGCGGAGCTGCTGCTCAG

>dogHBM_intron1

gtgggctgcggcgggggcgcgggccggggtgcctgggggcgctgggggcgggggccggggggtacccggggtgcccggggtggggggcggccgtcctcaccggcgcccggcgcgcag

>dogHBM_exon2

GCTCTTCACCGTGTACCCCAGCACCAAGACCTACTTCCCGCACCTGGGCTCCTGCGCCGACAAGGCGCAGCTGCTGAGCCACGGGCGGCGCATGCTGGAGGCGGTGGGCGTGGCCGTGCAGCACCTGGACAACCTGCGCGCCGCGCTGAGGCCGCTGGCCGACCTGCACGCCCAGGTGCTGCGCGTGGACCCCTCCAACTTCCCG

>dogHBM_intron2

gtgagcgcgccgcggcctcgggggggtgggggtgggggggcagcagggcccgcggaggcagcgccctcaccagcacctgtgcccccgcag

>dogHBM_exon3

CTGCTGATCCAGTGTTTCCAGGTGGTGCTGGCCTCCCACCTGCAGGACGAGTTCACCGTGGAGATGCAGGCGGCGTGGGACAAGTTCCTGACGGGCGTGGCGGTGGTGCTGACGGAGAAGTACCGCTGA

>dogHBM_3’UT500

gcccgctcccccgcccacatccccgcgtgtcaataaacacgggtcaagcggcaccggcgcctgtgtgctctgtgggctgcgggcgggcacctcggggcaccttagggcaccaccccaaccctcgcgcaccagggacaggggcccgggactctgaaatggggacagttccttggttatagtcccgggtctgggcagggtgtggatccccaaagccttggacagaaccgttgccgaggggcgatggggcaatgaggggggcaccgggggcgctgacatgagggagccctggccgggtgctctcacacagcgacaccgttcacaaagcacttgtgtgcgttcgctccctcctggaacctgcctccatccttcccgctgggcgctaccagcttgaattctcacgtcttttgctgacaaataccagcaccgcccacccccaatggccaagttcctgggctcaggtcaaaccggttgggactcctcaaggcctttgcttcccccca

>dogHBA1_5’UT500

cgcctgcaccagtcacctggcgctgcccaggagcgcctgcccagcttggggcaaagagggcttgaaggaccacataatctccctcaccaccactctttacatggacaatcgacagctctccatcctggtggatgtttggggcgaaagcagcttgttcacatcagaaaatattgtgctcccctccccagtcggcaaataccctagtttcagagccaaaagcagggggtggggcggttcgggaccccgtggtgggctcccgggaaccacaggagccagaagggagggtgccacggggagctgagctcggtctgctcaggacacaggacagagagtgagggcggccttgggacccccccacctgctccaggacctcggggcactgggccagccaatgagcgtggccccgatgggtgtgcccccccgcggccctggcataaagcctccgaggcctcccggccccgcacatttctggttctcctcagactcagaaggaacccacc

>dogHBA1_exon1

ATGGTGCTGTCTCCCGCCGATAAGACCAACATCAAGTCCACTTGGGATAAGATTGGTGGCCACGCTGGCGACTACGGCGGAGAGGCGCTGGACAG

>dogHBA1_intron1

gtgagcacccagctctccccggcgctggggctcggcggccccccgcggcccctgtccccacccccgcagcccctgtccttccccagcacctgtccccctcctgtggcccctgtccccacccccgcggcccctgtccgcctccccccgccccaccctctgccccggtccttacttcatggcccctgtgcccctcccacagtccttgtcccaccccacccctgtccccctcctgcgccccttgtccaccccccgtggcccctgtcccccccccagtggccccagtccacctgctggggcccctgtccccttgcccagcgcccccctccgtggcccctgtcctccccccgcagcccctgtcccttccctgcggcccctgtccttaacccatggcccccatccccctcctgtggcccctgttcccccacccccacccctgtccccctcctgcggcccttgtccccccccccgcagcccctgtcccccccaccagcggccccagtccacctcctggggcccctgtccccttgcccagcaccccctccgaggcccctgtccacagcccctgtccttactccctgtccccccacacccctgtccccctcctgcggcccttgtcctccccccacgaccccccgccgcggctcactggcccttcctcccacag

>dogHBA1_exon2

GACCTTCCAGTCCTTCCCCACCACCAAGACCTACTTCCCGCACTTCGACCTGAGCCCCGGCTCCGCCCAGGTCAAGGCCCACGGCAAGAAGGTGGCTGATGCGCTGACCACCGCCGTGGCCCACCTGGACGACCTGCCCGGTGCCCTGTCCGCCCTCAGCGACCTGCACGCCTACAAGCTGCGCGTGGACCCCGTCAACTTCAAG

>dogHBA1_intron2

gtgagccgcgggccaggccaggacacacctgggggcggcgcacggagacccccagagctccctgtggtgcagcccgcccccccccctgacggcccctccctctgcag

>dogHBA1_exon3

CTCCTGAGCCACTGCCTGCTGGTGACCCTGGCCTGCCACCACCCCACCGAATTCACCCCTGCCGTCCACGCCTCCCTGGACAAGTTCTTCGCCGCCGTGAGCACCGTGCTGACCTCCAAGTACCGTTAA

>dogHBA1_3’UT500

gctgcagccatggcggcgggagcccctgccctgggcccctgggtggcctcctgcaaccgtgccctacacttcctgatgtttgaataaagtctgagtgggctgcagcctctcaaggcctccgttgtctttgtctgcgagcagtgcgggtggtctcctccccgcaggggacctctgccggccttagtggctcttggaactaaagctgaggcttcctggaatgagcggagtcatgctccattcccagaaggtgccaggcttccagggttcctctctggagtgacaagcaggggaggctaagactgccaccctttttcttttctttttttttttttttttttaagatttatctatttatttatttattagagagagagagagggtggggaggaagagggacagggacaagcccactgagcactgagctccaagctgtggtggggctccatctcatgaccctgaggctacgatccgagcagaaaccaagagtaggatgcctgact

>dogHBA2_5’UT500

agaattcgtggggaggaggggttaggaggagcgtgtctgaggggagcaggaggggctccaggtgccaattccggagttggcgggagggaggcctgtgtgcacctctctccgccccagaccaggcaggccttttgtccgccctcaaccgctcagtctttcacggactccgctcgagttaacccagacgggctgcgagcgccaagcctacaacagggcaggtgtcccttaggatcctcaggagcacgcagcggggggctaggggcgctcgggcaccctcggggtgtccctgagcccccctgcaccgacccccaccagccggggccctggcggtgctagcggggctggagaagtgggcaggagagaacctccagtcagttggccccagcgagccccccacacccacccccgccagccaataagcgtggccccgacgggcgtgcccacccgcgccccgggcataaagcctccacggcctccccgccccgtgcatttctggttct

>dogHBA2_exon1

ATGGTGCTGTCTCCCGCCGACAAGACCAACATCAAGTCCACTTGGGATAAGATTGGTGGCCACGCTGGCGACTACGGCGGAGAGGCGCTGGACAG

>dogHBA2_intron1

gtgagcacccagctctcgccggcactggggctcggccgccccccgtgccccgtgtccgcctccctgggcccctgtcctccccccagacccctgttattcccccacggcctcttgtcctacccccgcagcccttgtcctccccccgtggcccctgtacttcccctatggcccctgtccttcccccggggcccctgtccccactcccacagcccctgtcccccctcctgcggcccttgtcctccccccgtggcccctgtcctccccccgcggcccctgtcctcctcccgtggcccctgtacttcccctacggcccctgtccttcccccggggcccgtccaccccccaccctgcccctgtccctctcctgcggcccttttcctccccctggggcccctgtccccccccccacccccgccgcatcaccggcccttcctcccgcag

>dogHBA2_exon2

GACCTTCCAGTCCTTCCCCACCACCAAGACCTACTTCCCGCACTTCGACCTGAGCCCCGGCTCCGCCCAGGTCAAGGCCCACGGCAAGAAGGTGGCTGATGCGCTGACCACTGCCGTGGCCCACCTGGACGACCTGCCCGGTGCCCTGTCCGCCCTCAGCGACCTGCACGCCTACAAGCTGCGCGTGGACCCCGTCAACTTCAAG

>dogHBA2_intron2

gtgagccgcgggccaggccaggacacacctgggggggggggcacagagacccccagggctccctgcggtgcagcccgcccccccctgacggcccctccctctgcag

>dogHBA2_exon3

CTCCTGAGCCACTGCCTGCTGGTGACCCTGGCCTGCCACCACCCCACCGAATTCACCCCTGCTGTCCACGCCTCCCTGGACAAGTTCTTCACCGCTGTGAGCACCGTGCTGACCTCCAAGTACCGTTAA

>dogHBA2_3’UT500

catggtgccctgcacttcctgatgtttgaataaagtctgagtgggctgcagcctctcaaggcctccgttgtctttgcgagctgtgcgggtagtctcctccccgcaggaggggggggttggggggtggaaagcatagggcagctgaggggagtgtggctggcctcccctggggactgccactgcctgggaatcgcaggcctgctccctggccacacccaggagtcctcagctccctttatttgatgtcctgcatttcacttaagagctttgcagattcagttgggatttgctcaaatgatgaactggtttccatctttatttttttttctaaatatttatttattcattcagagagagcaagagagaggcagagacacgggcagagggagaagcaggctccatgcaggaagcccgatgtgggactcccgctgcgccaccggggctgccctggtttccatctttacatttgaagacacacacttggtctgagagttgatgcg

>dogHBE_5’UT500

ATAATGCACTCCTCAAGTGAGTGAAAACTGGCTGAGATTCTATGTCAGCAACTGTTCTTTTTTTTTTTTTTTTTTTTTTCATGACTCTGTTCTTGTCTCTATCACCATCTTTAATCCAAATATTGAAACTGTTCTGACTACAACTCTTTCCTTATCTTGGGCTTAGCTCCTTTCTGCAGGTCTTTCTTGGAGAAGAAGAAAGGGAGAGATGGATGTCATTTTGGAGGATCATGTTTAGGACAGAGGAACCAACAAAGGAAGCCTGTCTCAGGAACCAACACACATTATCACAAGTTCAGTGGACATGCTTCACTGCTGACCCTTCCCTGATCTGACTCCACCCCCGAGGGACACAGTTTAACCTTGACCAATGACTTTAAAGGACTAGGAGGAACAAGGGGCCAGAAGGTCAGCAGTAAAGAATAAAAGGTGACAGTTTCCAGCAGCAGCACATACCTGCTTCTATCTCTGATCGCCTGTAAGCTCTCAGACCTGACATC

>dogHBE_exon1

ATGGTGCATTTTAGTGCCGAGGAGAAGGCTGCTATCACCAGCCTGTGGGGCAAAGTGAATGTGGAAGATGCTGGAGGCGAGGCCCTGGGCAG

>dogHBE_intron1

GTAGGCACTGGCTTTCAAGGCATGAGAAAGGGTGGTGAATATGAACCTGGCCAATTGACCAGGAAATTCCTCAAGATTTTTGCAAGTCTCTGATTTTCCATCTTCTGTGGTTCTGTCACATAG

>dogHBE_exon2

GCTCCTGGTTGTTTACCCCTGGACCCAGAGATTCTTTGACAACTTTGGCAACCTTTCCTCTGCTTCTGCCATAATGGGTAACCCCAAGGTCAAGGCCCATGGCAAGAAAGTGCTGACTTCCTTTGGAGATGCTGTTAAGAACATGGACAATCTCAAGGGCACCTTTGCTAAGCTTAGTGAGCTGCACTGTGACAAGCTGCACGTGGATCCCGAGAACTTCAGG

>dogHBE_intron2

GTGAGCTCAGGAAATGCTCATGTGCTCTTTTATTACTTTTCACTCTGGGGTAATAATGGAATTTGAGTCTTTGGAAAAGCTAACAGAAATTTCAGAAATTATGGGTCGAACTTGGTGTTAGAAGGACAGATCTCTAGTGGGCATAACCAGGACCACTTGGTTCAGGACTAGTTATAGAAGGGCTAGTAGCAGCCAAAATTTGCTTAAATCTTTTTAAGAACTTTTGTCAGAACTGGATATGTTCATCATAGAGAATGTTAGACTTGTTCAAGGAGGAATAGGATTTGGTGTTTGGTAGATAAAGGCCTTTCTCAAGGAAGGGGAAATGTCTTATTTTATGTGGTTTCGATGACTAAAGTTTAGAGAGAGATTTGGGGGGGAAATTTTAGACAGACTGTCTCAAAGTAATATACAAGTTCTTTAGAATATATTAAATTACCTGTCAGTATTTTTACCGAGTGGAGTTTTGTTCTTGCACTGATTGGAGACTTAAATAAGCTCTCTCTGGGGACCCCTGCAGCCCTAAGATCCCATTATTACAGGAATTAGCCAATTGGTACATAAGAGAGGGGAGGAGAGATGCAAATAAAAAAATAAAAATGGTGGCCTGGTAGAGAGGATGGGAATATGGGGAGGCAGGGAGTTAAATGGAAAGCTCACACTCATTGAATTTAAGGACTAGCTTATTTGGGCTTTTGCAGGCATACCTCCTGGAGCAGTTTAAGAATGTAGAATTGGTAGGAAGTAGAGGTCTGAGCTATTTTTTCTGCAATTCTTTTTGAGAACTTCTACTCAACATGCCTATTTGTCATTTTTGTCTTTCACCTAACAG

>dogHBE_exon3

CTCCTGGGCAATGTACTGGTGATCATCCTGGCTTCTCATTTTGGCAAGGAATTCACCCCCGATGTGCAGGCCGCTTGGCAGAAACTGGTGGCTGGTGTTGCCACTGCTCTGGCCCACAAATACCACTGA

>dogHBE_3’UT500

GTCCTCTTTCTAGTTCAGCAGTACCCTTCTGTGTCCCCAGTGTCCTCCTCCTGCATATGGGGACTTAGGTTTAGCTTTGAGAGCACAACTTCTGTTTAATAAAGTTCATTCTAATCAGTAATCGGAAATTAATATTGTGTCTTCTCCATTTTTTACTCTTGTGTTAAAGGAGAAAAGTTTCATTGACTGAGGGTTAGGAGACATGGGAAGAAACAAGAGTTCCTTTGGAAAGATAGACATTATGGGAGGATTCTAGAGGATCTGGGTGGATGCCAGGAGGGGGAAGAAATTTTCTGGGAAAGGCTTAATCAATTGGGTAGGGATTTACAGGGCAGGATGGCAAAAATGTACTGTAGAGGGGAAAAAAGGAGGTGCTAAGCAGTGCCTCTGAAAAAACAAGCTAGATTTGTGGTACATGTCAGACTTCAGCTTTAAAACCTTTTCAGGGATCTAAGGTCAATTTGCTTTAATGTACATATTGAATCAGCTAACTTATCATA

>dogHBH_5’UT500

TATTTCTTTTTACAAGAACTATAAAATTATGAGCTTCATCTAAGTTTTAGAAAAACTGGTGACCAAGAAAAACTATGGCTTCTTTTATACCCAAGGTGAACAACAGAATAATACTTTGTAAACACCTGACACAATGCCCAACAGATTTCTAATTCTTACCTCTCTTAATTCTAGTTATGCCTCATGATCAGACTACAAGGTCACCTCAGGAATAATGGGGTGGGGAGCTTGAGATATGAAATGCATAGTAGATGGTGGAGAAAGGACGGTAGAATCCCCCTGATAGAGCCACCCAACGCTATCAGAGACCCAAATGTCCATCCTGCTGACCCTCCCTTGTCCAAACCCCACCCCTGGTGGTGACAACCCACTCTTGACCAATGGTCTCATTTCACTGGGAGAAGCAAGGGCCCTGAAACTAGAAATGAGGAATAAAAGGCCACGCAGAGAAGTGGCAGTACAGACTTGTTTCTGGCACTTTAGTGATCACCAGCAAGTTC

>dogHBH_exon1

CCAGACACCATGGTGCATTTTACTGCTGAGGAGAAGGCTGCTGTTATTAGCCTGTGGGCCAGGGTGAATGTGGAGTTGGTTGGAGGCGAGGTCCTGGGAAG

>dogHBH_intron1

GTAGGCACTGGAGGGGGGCAAAAAGGCAGAAAATATTCCTGAAAGGTGTTTGGTCAGGTTTCCTACATACTCTGACTTCCCATCTGGTCTGTGACTGTGATCATCCCATAG

>dogHBH_exon2

GCTCCTGGTTGTTTATCCATGGACCCAGAGGTTCTTTGACAATTTTGGCAATTTGTCCTCTGAGTCTGCAATAATGGGCAACCCCAAGGTCAAGGCCCATGGCAAGAAGGTGCTGACTTCCTTTGGAAATGCTATTAAACATATGGATGACCTCAAGGACACCTTTGCTGAGCTAAGTGAACTGCACTGTGACAAGCTGCATGTGGATCCGGAGAACTTCAAG

>dogHBH_intron2

GTGAGTTCTGGGCATGCCTGTGCTTTGTTCTTTCATCCTAGGTATTTGTACTGTGGATATGTTAGGAAAAACAGGTACCTAAATCTGATCCTATAAAGGCATTTTTTTTGGGATGTTGTATCCCAAGGAAGGGCTTGATTTTGGTAGTTAGGTTGGCCCAAAGGTCATAGGTAGGACTCAATAGGGTAATTACTATGACCAGCGAAGGTCACTGGCCATCTTACATTTTTGAAAAGATACATATGTGAAGAACACTGGCTAAAATGGAGTTAAATGGCTTTGGTCAGGAGAATACAATTAGAGGAAGGACATCTTAACTGATATTTTCTCTAAAAATGATGAATACATCTCTGCATGATTCTTAAATTTAAAACCAGGACTAAGCAAGACAAAATAAGAGCAATCTTTTCAATGAACGATTGTGACCCCCCCTCTTTATTAGTGATGAAATTCTCTTATATACGTGGCACAAGATTCTTTGTCATGTGAATGTCCAAACAATTTGAATGAATGTATTATCTAGGGACATAGATTATACATATTAAGGCCCCTTGCAGTCCTACATTCCTACTTTTAAAGAAGTCAGAGTCTCTAGAAAAGAGAAGCCATCCATGGCAATGTTCTGGGGTCAGAAGTTGGCAGGCCAAATGAGAGGATAGAACGTAATGGAGTGTACAGGGCTGGGGGTCATATTGATGCCAATGTCCAGCATATTAAACTTTTGTGGGAGCCCAGCATTGGAAGTTCCTCCATCTCTTATTAGGAAGAAAATAGATGCGGCCAGCTTGAGGCTTTCCGTTCACTAGGTACTATTTTCTTTTTACCTCTCCTCTCTCCCCAG

>dogHBH_exon3

CTTCTAGGCAACATGATATTGATTGTCTTGGCAACTCACTTCAGCAAGGAGTTTACTCCCCAAACACAGGCTGCGTGGCAGAAGCTGACAACTGCTGTGGCTAATGCTCTGGCACACAAGTAC

>dogHBH_3’UT500

CACTAGGTGCTGGTCCAATTATGTCGGTATTGCCAGAGTCCATCTCCTGAAGACAGGCACAGTGCCTTGCCTAGATGATAAAAATAAAATAAAAATTGTGCTCTGTAATGAAAATCCTTGTCTTCTCTCTCTGTCTGTACCTTTTATGTTGATTCAGCTAAAAGAAAGCACTCCTACAGGAAAGGGGGTTACTGGAGATAAGGAGTTTGGGAGGATAGAGAAGAGACTCCTAAGATTCTGCAGGATAAGAGTGGGTTCAAGGGGCAGTTACTTCTAGGAAGAGAAAGTAGACTCTTGAGTTGAATAGTGTCTCTAGAATAAGGGAATGAATGGACATATTCTTGTGAAATTCAAGAAGATTGTTGAGAGTGAGTAGGGGCATGTTAAAACCCCAAGTAGGGGGCCTGTCTTGGCTGCTATTTGTATAATCTCAGAGCTTTTTGGTGCTGTATTTAGTTTCTATTTGATATAAGAAACATGTTACTGTATGTTGTCTGATT

>dogHBD1_5’UT500

AGTATTTATTCATTCTAACTACTTATAACTATCTCTAGAGACCAAAAGAAGTACCACATAGAGTCAACTTGATAGCATTTCTGTTTACTAGGCCTTAGTTTTCTTATCTATGAAGTGAAATAACTGGAGGAGTTAAACAAGAGATATACTCCATTATATCAGACTACAGAATTTGCACAAAAATGAGAGTGGGAGTATACTTATGGAAGCCACTGCTGAAGGGGATGAGGTGAGGAGAGGAAACAGGGATTCTGAGTCAAGAAACATAACACCTAGAACGCCACTCTCAGGACAATTGAGGGGAACTGAGGAATTGGGTTGCAAACT

>dogHBD1_exon1

ATGGTGCATCTGACTGCTGAAGAGAAGAGTCTTGTCTCCGGCCTGTGGGGCAAGGTGAATGTGGACGAAGTTGGCGGTGAGGCCCTGGGCAG

>dogHBD1_intron1

GTTGGTATCCATGTGGCAAGGCAGGCTTTTGGAGAAAACATGAGAGCTGGGCAGATGGAGTTAACTCAGTCCCTTGGGTTCTGACGGGCACAGCCCTCTTGCTGTTTTCACTCCTCAG

>dogHBD1_exon2

GCTGCTGATTGTCTACCCCTGGACTCAGAGGTTCTTTGACTCCTTTGGGGACCTGTCCACTCCTGATGCTGTTATGAGCAATGCTAAAGTGAAGGCCCATGGCAAGAAGGTGCTGAACTCCTTTAGTGATGGCCTGAAAAACCTGGACAACCTCAAGGGCACCTTTGCTAAGCTCAGTGAGCTTCACTGTGACAAGCTGCACGTGGATCCCGAGAACTTCAAG

>dogHBD1_intron2

GTGAGTCTAGGATATGTTCCATTTTTTTTTTCTTTTCACTTTCTAGTCTCTCACCTGATTCATTTACCTACCTGTTCTCCCTCCACCTTCCTTTGTACTTCAGCATATATCATCTTGAATACCTTTCAAAGTTTATGTAATTTCATACATTTCCTTTCTCACTGCCTTTTCTTTTTCATCAAGTTATTTATTTAACTTCTTGTCTTCTTCCCCTGACTAGTTTTTTTTCCCTACTCGATATGCAAATTATGCGTGTCTACTCTCATCTACTTCTCCACTCGGAAACATCTTTCTGTCTCTCCAAATGGGGATTGGAAGGGCACCTCAAGGAGTTGTCAATCTAGAGGCTACAAATCATTTCAAAATTAAAGGATAATTGGATTTTTATAGAGACAAGACTCAATGGAAAGGAAAGGAATTGAATATCTGAGAATAGTGAAGTAGGACACCCACAGGTGCTAAAAGACAGCCCAGCATCATACTAATTAATCAATTAACTATTAAATTatatatatatacatatataatgaaaatatacagtgtacatacgcatataTTTGCTCATTTATGCTGATGGAGAAAACCTGAAAATCAGTTTGGGCTGAAGTGTGGGGAAAAAAAAAATCATTGGCTCAGTTTCTCAGAAGCCGAGTTTGATTTCTCTCTTAATTGTGTATGCATGTCTCTCTATGTCTTCCCCACAG

>dogHBD1_exon3

CTCCTGGGCAACG

TGCTTGTGTGTGTGCTGGCTCACCACTTTGGCAAAGAATTCACCCCTCAGGTGCAGGCTGCCTATCAGAAGGTGGTGGCTGGTGTGGCCAATGCCCTGGCTCACAAGTACCAT

>dogHBD1_3’UT500

TGAGATCCTGGCCTGTTTCCTGGTGACCACTGGAAGACCCTGTCTCCCTAAATTCTTTCTTCTGAACTGGGGGAAATAATGTCCACCATCAAGGGTATGGCTTCTGCCTAATAAAGAACCTTCAGCTCAACTTTCTGATTCATTTCACttattttattttatattttattttattattattttttactttatttGTCTAGGGGTGTGGGAAATACTTGAGAGTCTACAGATAGGAAGCACTTGTTTCTTATTCAAAGAGGTCAAGGGAGATGAGAAAAGGAAGTAGGGGCCTACATTGTCACTAGTGGAAAACACTTCTCCCTCCAAAACATAGGCACGGTCAAGAAGATTTTATATAGAGAATGCTGGCATTATAGGGGCTCTGTGGAAGACTTCCAACTTAAGGCACAGCCCTCAGGCTAATGCTTTTACTTATTTCCAACAATTAGTTAAAAAACAGGATGCTCGATTAGAGACATAATGAATGATCAAGATACTGT

>dogHBD2_5’UT500

TCCAAACCAAGAGAAGGAAGATACACGTAGGACTGTGGCAGAGGTCCCATCCACACTGTCCATGTAATTAGATTCTGAAGACACAGGGGGAGATCCATCCATACGCTTTTAAGTTGAACACAGTGGATGGATGCCTTCTATTTActggacctgggattcttcatttgtataacaagaaaattggggaggcactctctaagAAGTTACCAGGTTATGGTTCTCAAAAGTGTAACAGTACACTTGCCAAAGAATGATTTTAGTAGCAATTTGTATTGCTGAGATGGGCCTGAAGATGGGAGGGTCTGAAGTCAGACTCACAAGCCAGTTTCAGAATTGCCAAGGATAGGCTTCACCCTGCGTGACCACACCCAGGCCTGGGCCAATCTCATAACAGCAAGGATGCAAGATCAGGGCTGGGCATAAAAGGAAGAACAGGGACAGCTGCTGCTTACATTTGCTTCTGAAACAACCGTGTTCACTAGCAACCACAAACAGACACC

>dogHBD2_5’UT250

ATGATTTTAGTAGCAATTTGTATTGCTGAGATGGGCCTGAAGATGGGAGGGTCTGAAGTCAGACTCACAAGCCAGTTTCAGAATTGCCAAGGATAGGCTTCACCCTGCGTGACCACACCCAGGCCTGGGCCAATCTCATAACAGCAAGGATGCAAGATCAGGGCTGGGCATAAAAGGAAGAACAGGGACAGCTGCTGCTTACATTTGCTTCTGAAACAACCGTGTTCACTAGCAACCACAAACAGACACC

>dogHBD2_exon1

ATGGTGCATCTGACTGCTGAAGAGAAGAGTCTTGTCTCCGGCCTGTGGGGCAAGGTGAATGTGGACGAAGTTGGCGGTGAGGCCCTGGGCAG

>dogHBD2_intron1

GTTGGTATCCATGTGGCAAGGCAGGCTTTTGGAGAAAACATGAGAGCTGGGCAGATGGAGTTAACTCAGTCCCTTGGGTTCTGACGGGCACAGCCCTCTGTGCTGTTTTCACTCCTCAG

>dogHBD2_exon2

GCTGCTGATTGTCTACCCCTGGACTCAGAGGTTCTTTGACTCCTTTGGGGACCTGTCCACTCCTGATGCTGTTATGAGCAATGCTAAAGTGAAGGCCCATGGCAAGAAGGTGCTGAACTCCTTTAGTGATGGCCTGAAAAACCTGGACAACCTCAAGGGCACCTTTGCTAAGCTCAGTGAGCTTCACTGTGACAAGCTGCACGTGGATCCCGAGAACTTCAAG

>dogHBD2_intron2

GTGAGTCTAGGATATGTTCCATTTCTTTTTCTTTTCACTTTCTAGTCTCTCACCTGATTCATTTACCTACCTGTTCTCCCTCCACCTTCCTTTGTACTTCAGCATATATCATCTTGAATACCTTTCAAAGTTTATGTAATTTCATACATTTCCTTTCTCACGGCCTTTTCTTTTTCATTAAGTTGTTTTTTTTTTTCATCAAGTTGTTTATTTAACTTCTTGTCTTCTTCCCCTGACTAGTTTTTTTCCCTACTCGATATGCAAATTATGCGTGTCTATTCTCATCTTCTACTTCTCCACTCGGAAACATCTTTCTGTCTCTCCAAATGGGGATTGGAAGGGCACCTCAAGGAGTTGTCAATCTAGAGGCTACAAATCATTTCAAAATTAAAGGATAATTGGATGTTTATAGAGACAAGACTCAATGAAAAGGAAAGGAATTGAATATCTGAGAATAGTGAAGTAGGACAGCCGCAGGTGCTAAAAGACAGCCCAGCATCATACTAATTAATTAATTAACTATTAAATTatatatatatacatatacaatgaaaatatacagtgtacatacacatataTTTGCTCATTTATGCTGATGGAGAAAACCTGAATATCAGTTTGGGCTGGAGTGTGGGGAAAAAAAAAAAAAGATCATTGGCTCAGTTTCTCAGAAGCCGAGCTTGATTTCTCTCTGAATCATTTATGCGTGTCTCTCTATGTCTTCCCCACAG

>dogHBD2_exon3

CTCCTGGGCAACGTGCTTGTGTGTGTGCTGGCTCACCACTTTGGCAAAGAATTCACCCCTCAGGTGCAGGCTGCCTATCAGAAGGTGGTGGCTGGTGTGGCCAATGCCCTGGCTCACAAGTACCAT

>dogHBD2_3’UT500

TGAGATCCTGGCCTGTTTCCTGGTGACCACTGGAAGACCCTGTCTCCCTAAATTCTTTCTTCTGAACTGGGGGAAATAATGTCCACCATCAAGGGTATGGCTTCTGCCTAATAAAGAACCTTCAGCTCAACTTTCTGATTCATTCCACttattttattttatattttattttattattattttttactttatttGTCTAGGGGTGTGGGAAATACTTGAGAGTCTACAGATAGGAAGCACTTGTTTCTTATTCAAAGAGGTCAAGGGAGATGAGAAAAGGAAGTAGGGGCCTACATTGTCACTAGTGGAAAACACTTCTCCCTCCAAAACATAGGCACGGTCAAGAAGATTTTACATAAAGAATGCTGACATTACAGGGCTCTGTGGAAGACTTCCAACCTAAGGCACAGCCCTCAGGCTAATGCTTTTACTTATTTCCAATAATTAGTAAAAAATAGGGTGCTCGATTAGAGACATAATGAATGATCAAGATACTGTTC

>dogHBB_5’UT500

CCAAACCAAGAGAAGGAAGATACACGTAGGACTGTGGCAGAGGTCCCATCCACACTGTCCATGTAATTAGATTCTGAAGACACAGGGGGAGATCCATCCATACGCTTTTAAGTTGAACCACAGTGGATGGATGCCTTCTATTTActggacctgggattcttcatttgtataacaagaaaattggggaggcactctctaagAAGTTACCAGGTTATGGTTCTCAAAAGTGTAACAGTACACTTGCCAAAGAATGATTTTAGTAGCAATTTGTATTGCTGAGATGGGCCTGAAGATGGGAGGGTCTGAAGTCAGACTCACAAGCCAGTTTCAGAATTGCCAAGGATAGGCTTCACCCTGCGTGACCACACCCAGGCCTGGGCCAATCTCATAACAGCAAGGATGCAAGATCAGGGCTGGGCATAAAAGGAAGAACAGGGACAGCTGCTGCTTACATTTGCTTCTGAAACAACCGTGTTCACTAGCAACCACAAACAGACACC

>dogHBB_exon1

ATGGTGCATCTGACTGCTGAAGAGAAGAGTCTTATCTCCAGCATGTGGGGCAAGGTGAATGTGGATGAAGTTGGCGGTGAGGCCCTGGGCAG

>dogHBB_intron1

GTTGGTAT

CCATGTGGCAAGGCAGGCTTTTGGAGAAAGCATGAGAGCTGGGCAGATGGAGTTAACTCAGTCCCCTGGGTTCTGACAGGCACTGACTCCTGTGCCCTCTGTGCTATTTTCACCCCTCAG

>dogHBB_exon2

GCTGCTGATTGTCTACCCCTGGACTCAGAGGTTCTTTGACTCCTTTGGGGACCTGTCCACTCCTGATGCTGTTATGAGCAATGCTAAAGTGAAGGCCCATGGCAAGAAGGTGCTGAACTCCTTTAGTGATGGCCTGAAAAACCTGGACAACCTCAAGGGCACCTTTGCTAAGCTCAGTGAGCTTCACTGTGACAAGCTGCACGTGGATCCCGAGAACTTCAAG

>dogHBB_intron2

GTGAGTCTGTGGGACCCTCAACATTCTCTTTGATCTTCCTTTTTAAGACCCAACTCATGATGTCCGTAAAGGGTATGGGCATCAGGATGTTGTTCAGAGTGTAAAGGAAGTGTTCTAGTTGCATAAGTATGGACTCCTCAGCACTGTTTTACTTATTTTATCCACTTGGCTCATTAATTGTTTCCTCTTGTCCTTTTTTTTGTTCTCTGCAATGGTTTTTCTTTATTGTTTTAATTAAACTTTTGAGTGTTTAAAAAGTGCTTTTAATCCATTCAAAAACATTTTTTTTTCTGATCCTTTTTTCCTTATCTCTTTTTTGAAAGCAAGGAAGATAAAATCTTATTGCTTCTTTGCCTAGTTGGAAAGAATAACCAAGAAAATTTAAGATTTGGGTTAAAACAGAAGGAAAGAACCATTTCTCAATGTAAAATGAGGGTGATATTGTTGGAGTCATATGGATTGTAACATCTTAATAATCAGCTATCTTTCTGCTTATATTCTAATGACACTGTTTGGGTCTGTGGATGGGTTGGAATACTCTGAGTCTTAGCTAGGTCCCTCTGCTAATCATCCACATGCCTCTTGTCTTCCCCACAG

>dogHBB_exon3

CTGCTGGGCAACGTGTTGGTGTGTGTGCTGGCCCGCCACTTTGGCAGGGAATTCACCCCATTGGCTCAGGCTGCCTACCAGAAGGTGGTGGCTGGTGTGGCCAATGCCCTGGCCCACAAATACCAC

>dogHBB_3’UT500

TGAGTTCTCTTTCTTGCTTTCAAGGCAAAGTCATTTGGTCCCCGGACCCCAACACTTGAACATGGGGAAATTGCGAAGTCCTTTGAGCATCTGGTTCCTGACTAATAAAGAACATTTATTTTCTATGTACTGGTGTATTTAAATTACTTCTGTGACTGTCACTGGAATTGTTACATCACATGAGAAGTCAAGTCATGTAAGACATAAAGAATGAGGGGCTAGTTCAGATCTTGGGAAAATATATCCGTATTTTGGACTCCATGAATGGAGAGGTTATAAATAGCTAACTATGTGGCAAACTGACCCTGCTGCCTATTTTTACTTTCCCTTAGGAAAGGATTCAACTAGAGACTTGCTTTGGGGGTTAGGCTTTTCCTATGCTTT

ATTTAAATCAAGCATTTTATTTAGGCTTACTTAAATTTCCTCACTCTCCCCAGTTCAAAAACTCACTTTAATACGCAAAATTCTTCTGCCTAAAGGCATCAGTGCTCCCCTAAGAT
