# Supplementary figures and images for: Comparative genomics of canine hemoglobin genes reveals primacy of beta subunit delta in adult carnivores
Source: BMC Genomics. 2017 Feb 8;18:141. doi: 10.1186/s12864-017-3513-0 (PMC5299747; doi:10.1186/s12864-017-3513-0)

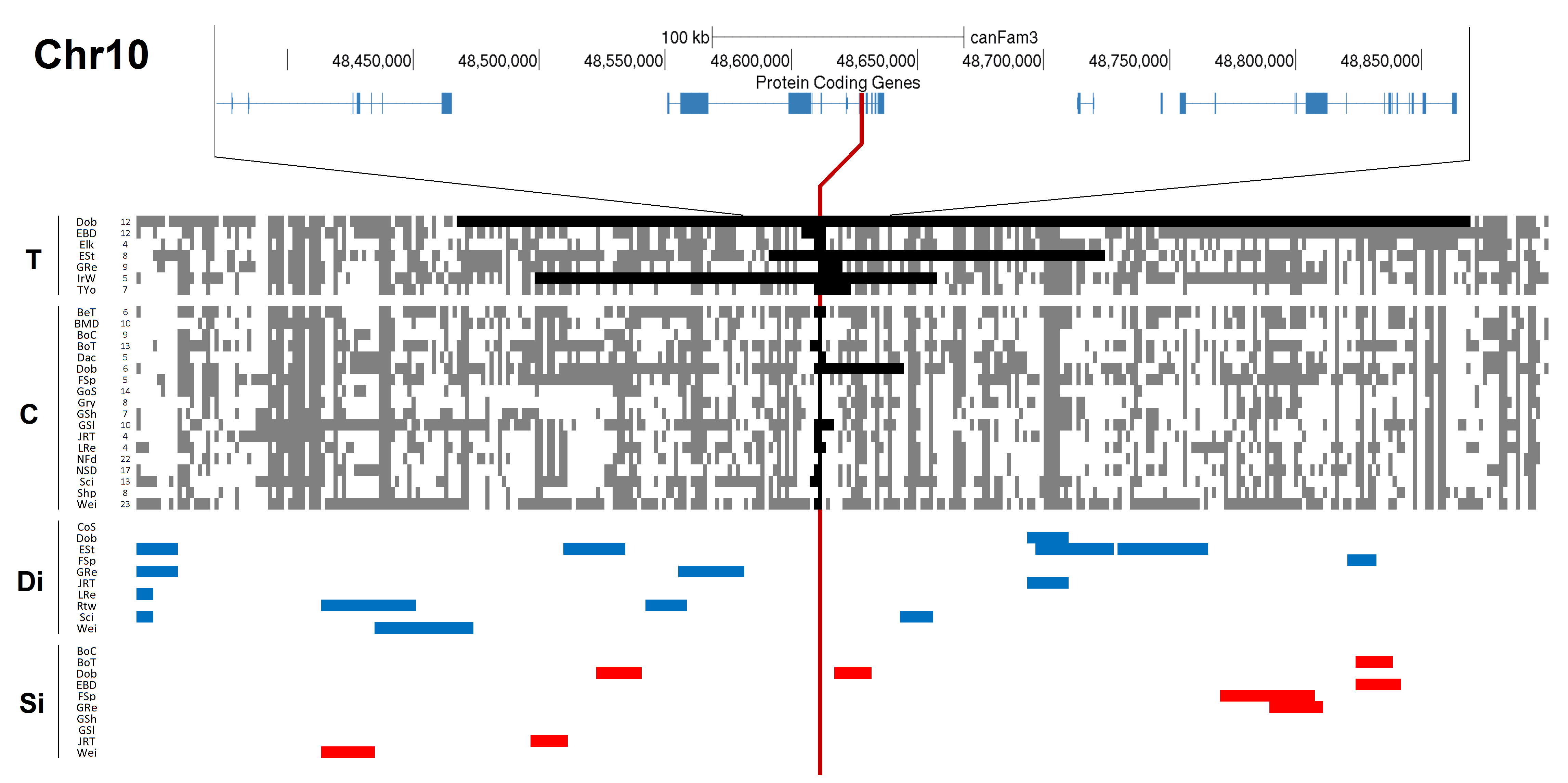

Supplement: Additional file 2: — EPAS1 (HIF-2alpha protein) gene region may harbor variation under selection. Direct haplotype phasing anchored within (shown here by vertical line) or near the EPAS1 gene show that three breeds have large (Irish Wolfhound and English Setter) or very large (Doberman Pinscher) phased haplotype blocks (shown as black horizontal bars). The latter two of those breeds also have evidence of population differentiation (D i statistic, blue bars), and the Doberman Pinscher also has evidence of reduced heterogeneity (S i statistic, red bars) [4]. (TIF 432 kb) [file 12864_2017_3513_MOESM2_ESM.tif]

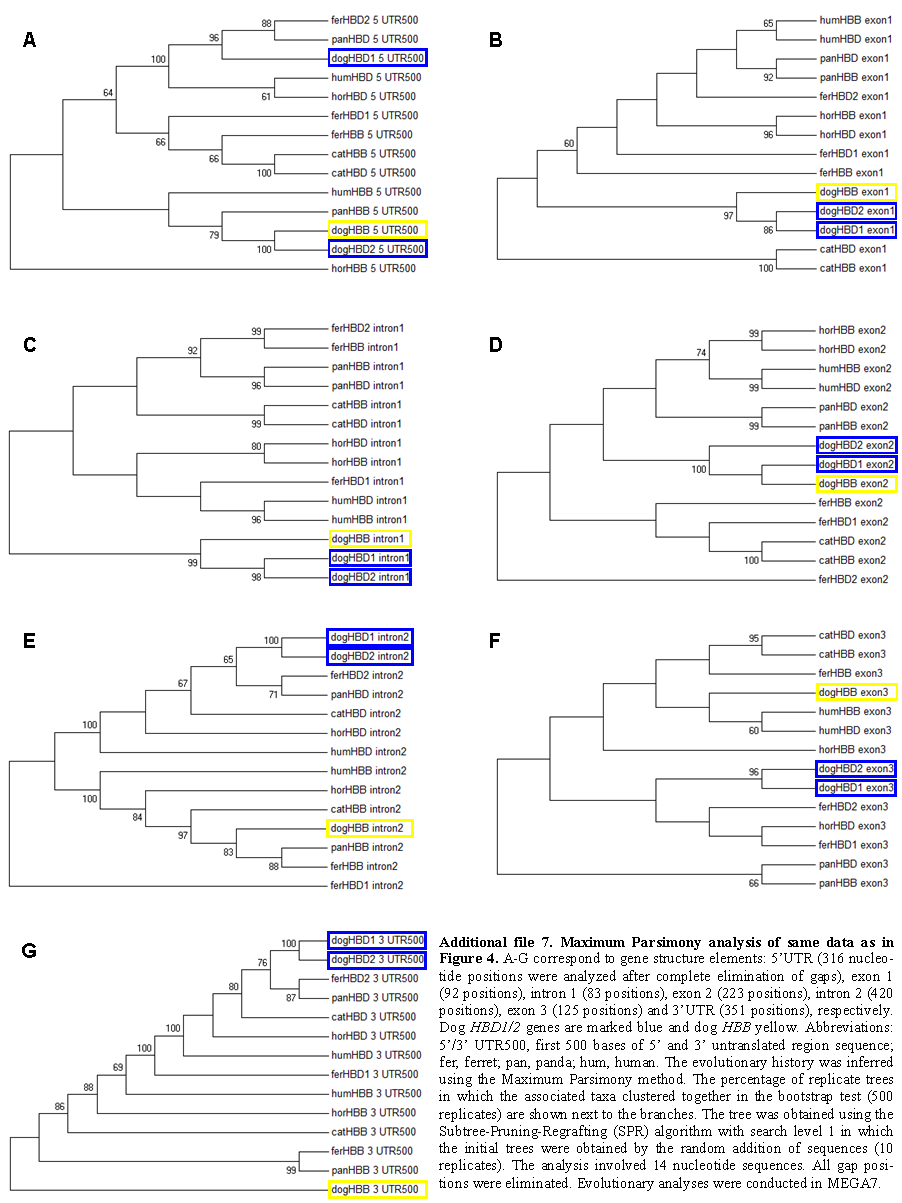

Supplement: Additional file 7: — Maximum Parsimony analysis of same data as in Fig. 4, phylogeny of HBD and HBD genes from dogs and select other species. (PNG 223 kb) [file 12864_2017_3513_MOESM7_ESM.png]
