# Supplementary material for: Comparative genomics of canine hemoglobin genes reveals primacy of beta subunit delta in adult carnivores
Source: BMC Genomics. 2017 Feb 8;18:141. doi: 10.1186/s12864-017-3513-0 (PMC5299747; doi:10.1186/s12864-017-3513-0)
Supplement: Additional file 3: — Complement of β-globin proteins in the domestic cat. (DOCX 14 kb) [file 12864_2017_3513_MOESM3_ESM.docx]

**Complement of cat beta globin proteins**

>catHBB_felCat5_chrD1 range 63868731-63870359 partial plus HTGS AC129836.3 range 131307 to 131399 length=147 (predicted in this work; Zaldivar et al.)

MSFLSAEEKGLVNGLWSKVNVDEVGGEALGRLLVVYPWTQRFFQSFGDLS

SADAIMSNSKVKAHGKKVLNSFSDGLKNIDDLKGAFAKLSELHCDKLHVD

PENFRLLGNVLVCVLAHHFGHDFNPQVQAAFQKVVAGVASALAHRYH

>catHBD_ENSFCAT00000016411 length=147

MGFLSAEEKGMVNGLWGKVNVDEVGGEALGRLLVVYPWTQRFFQSFGDLS

SADAIMSNSKVKAHGKKVLNSFSDGLKNIDDLKGAFAKLSELHCDKLHVD

PENFRLLGNVLVCVLAHHFGHDFNPQVQAAFQKVVAGVANALAHKYH

>catHBH_ENSFCAT00000022944 length=149

MVHFTAEEKAAVVSLWAKVNVELVGGEVLGRLLVVYPWTQRFFDNFGNLS

SESAIMGNPKVKAHGKKVLTSFGNAVKHMDDLKDTFAELSELHCDKMHVD

PENFKLLGNMILIVLATHFSKEFTPQVQAAWQKLTAAVANALAHRYH

>catHBE_ENSFCAT00000002482 length=148

MVHFTAEEKAAITNLWGKVNVEEAGGEALGRLLVVYPWTQRFFDNFGNLS

STSAIMGNPKVKAHGKKVLTSFGDAIKNMDNLKGAFAKLSELHCDKLHVD

PENFRKLLGNVLVIILASHFGKEFTPDMQAMWQKLVAGVATALAHKYH
