# Supplementary material for: Comparative genomics of canine hemoglobin genes reveals primacy of beta subunit delta in adult carnivores
Source: BMC Genomics. 2017 Feb 8;18:141. doi: 10.1186/s12864-017-3513-0 (PMC5299747; doi:10.1186/s12864-017-3513-0)
Supplement: Additional file 4: — A) Multiple sequence alignment of embryonic β-globins from model placental mammal genomes, and B) consensus sequence logo from those embryonic β-globins. (PDF 1094 kb) [file 12864_2017_3513_MOESM4_ESM.pdf]

A)

```
                                          10        20        30        40        50        60        70        80        90       100       110       120       130       140
                                           |         |         |         |         |         |         |         |         |         |         |         |         |         |
HBE_shrew            MVHFTAEEKAAVASLWGKVDVEVAGAEILGRLLIVYPWTQRFFDNFGNLSSVSAIMGNPKVKAHGKKVMTSFGEAVKHMDDLKDAFAKLSELHCDKLHVDPENFVLLGNMILIVLATHFGKEFTPQMQASWQKLTSAVASALAHKYH
HBE4_cow             MVHFTTEEKAAVASLWAKVNVEVVGGESLARLLIVYPWTQRFFDSFGNLYSESAIMGNPKVKAHGRKVLNSFGNAIEHMDDLKGTFADLSELHCDKLHVDPENFRLLGNMILIVLATHFSKEFTPQMQASWQKLTNAVANALAHKYH
HBE2_cow             MVHFTTEENVAVASLWAKVNVEVVGGESLARLLIVCPWTQRFFDSFGNLYSESAIMGNPKVKVYGRKVLNSFGNAIKHMDDLKGTFADLSELHCDKLHVDPENFRLLGNMILIVLATHFSKEFTPQMQAAWQKLTNAVANALTHKYH
HBH_dog              MVHFTAEEKAAVISLWARVNVELVGGEVLGRLLVVYPWTQRFFDNFGNLSSESAIMGNPKVKAHGKKVLTSFGNAIKHMDDLKDTFAELSELHCDKLHVDPENFKLLGNMILIVLATHFSKEFTPQTQAAWQKLTTAVANALAHKYH
HBH_bat              MVHFTAEEKAAVANLWAKVNVEVAGGEILGRLLVVYPWTQRFFDSFGNLSSETAIMGNPKVKAHGKKVLNSFGNAVKHMDDLKGTFSHLSELHCDRLHVDPENFKLLGNMIVIVLATQFNREFTPQMQAAWQKLTTAVANALAYKYH
N/A-1_microbat       MVHFTAEEKAAVASLWAKVNVEVAGGEVLGRLLVVYPWTQRFFDTFGNLSSETAIMGNPKVKAHGKKVLTSFGNAVKHMDDLKSTYSQLSELHCDRLHVDPENFKLLGNMILIVLATQFGREFTPQMQAAWQKLTTAVANALAYKYH
HBBbh1_mouse         MVHFTAEEKAAITSIWDKVDLEKVGGETLGRLLIVYPWTQRFFDKFGNLSSALAIMGNPRIRAHGKKVLTSLGLGVKNMDNLKETFAHLSELHCDKLHVDPENFKLLGNMLVIVLSTHFAKEFTPEVQAAWQKLVIGVANALSHKYH
HBG1_human           MGHFTEEDKATITSLWGKVNVEDAGGETLGRLLVVYPWTQRFFDSFGNLSSASAIMGNPKVKAHGKKVLTSLGDATKHLDDLKGTFAQLSELHCDKLHVDPENFKLLGNVLVTVLAIHFGKEFTPEVQASWQKMVTAVASALSSRYH
HBG2_human           MGHFTEEDKATITSLWGKVNVEDAGGETLGRLLVVYPWTQRFFDSFGNLSSASAIMGNPKVKAHGKKVLTSLGDAIKHLDDLKGTFAQLSELHCDKLHVDPENFKLLGNVLVTVLAIHFGKEFTPEVQASWQKMVTGVASALSSRYH
N/A-2_orangutan      MGHFTEEDKATITSLWGKLNVEDAGGETLGRLLVVYPWTQRFFDSFGNLSSASAIMGNPKVKAHGKKVLTSLGDAVKNLDDLKGTFAQLSELHCDKLHVDPENFRLLGNVLVTVLAIHFGKEFTPEVQASWQKMVAGVASALSSRYH
N/A-1_orangutan      MGHFTEEDKATITSLWGKLNVEDAGGETLGRLLVVYPWTQRFFDSFGNLSSASAIMGNPKVKAHGKKVLTSLGDAIKNLDDLKGTFAQLSELHCDKLHVDPENFRLLGNVLVTVLAIHFGKEFTPEVQASWQKMVTGVASALSSRYH
HBG_gibbon           MGHFTEEDKATITSLWGKVNVEDAGGETLGRLLVVYPWTQRFFDSFGNLSSASAIMGNPKVKAHGKKVLTSLGGAIKNLDDLKGTFAQLSELHCDKLHVDPENFRLLGNVLVTVLAIHFGKEFTPEVQASWQKMVAGVASALSSRYH
HBG2_macaque         MGHFTEEDKATITSLWGKVNVEDAGGETLGRLLVVYPWTQRFFDSFGNLSSASAIMGNPKVKAHGKKVLTSLGDAIKNLDDLKGTFAQLSELHCDKLHVDPENFRLLGNVLVTVLAIHFGKEFTPEVQASWQKMVAGVASALSSRYH
HBG1_macaque         MGHFTEEDKATITSLWGKVNVEDAGGETLGRLLVVYPWTQRFFDSFGNLSSASAIMGNPKVKAHGKKVLTSLGDAIKNLDDLKGTFAQLSELHCDKLHVDPENFRLLGNVLVTVLAIYFGKEFTPEVQASWQKMVAGVASALSSRYH
N/A_squirrel         MVHFTAEDKAAITSMWRKVNVEEAGGETLGRLLVVYPWTQRFFDSFGNLSSASAIMGNPKVKAHGKKVLTSLGNALRDMDDLKGAFSQLSELHCERLHVDPENFRLLGNMLVIIMAKHFGKEFTPEMQASWQKMVAGVASALAHKYH
HBEY_mouse           MVNFTAEEKTLINGLWSKVNVEEVGGEALGRLLVVYPWTHRFFDSFGNLSSASAIMGNPRVKAHGKKVLTAFGESIKNLDNLKSALAKLSELHCDKLHVDPENFKLLGNVLVIVLASHFGNEFTAEMQAAWQKLVAGVATALSHKYH
HBE1_squirrel        MVHLTAEEKNLISSLWGKLNVEETGGEALGRVLVVYPWTQRFFDSFGNMSSPSAIMGNPRVKAHGKKVLTSFGEAIKNLDNLKGTFAKLSELHCDKLHVDPENFKLLGNVLVIVLSAHFGKEFTPEMQAAWQKLVSGVANALGHKYH
HBG_bushbaby         MVHFTAEEKAIITSLWGKVNVEEDGGEALGRLLVVYPWTQRFFDTFGNLSSASAIMGNPKVKAHGKKVLSSLGEAIKNMDDLKGTFSHLSELHCDRLHVDPENFRLLGNVLVIVLAKHFGKEFTPQIQAACQKMVAGVATALAHKYH
Hbby_kangaroo_rat    MVHLNSEEKALINGLWSKVNVEETGGEALGRLLVVYPWTQRFFDSFGNMSSPSAIMGNPRVKAHGKKVLTSFGEAVKNMDNLKTTFAKLSELHCDKLHVDPENFRLLGNVLLIVLSSHFGKDFTPEVQAAWQKLVAGVATALAHKYH
HBE_armadillo        MVHFTAEEKAAVANLWGKVNVEETGGEALGRLLVVYPWTQRFFDNFGNLSSPSAIMGNPKVKAHGKKVLSSFGEAIKHMDDLKTTFAKLSELHCDKLHVDPENFRLLGNVMVVVLASHFGKEFSPDVQAAWQKLVAGVANALAHKYH
HBE1_sloth           MVHFSAEEKATVASLWGKVNVEEAGGEALGRLLVVYPWTQRFFDNFGNLSSSSAIMGNPKVKAHGKKVLTSFGDAIKHMDDLKGTFAHLSELHCDKLHVDPENFRLLGNVMVVVLASHFGKEFTPEVQAAWQKLVGGVANALAHKYH
HBE1_tarsier         MVHLTAEEKSSVTSLWGKMNVDEAGGEALGRLLVVYPWTQRFFDNFGNLSSSSAIMGNPKVKAHGKKVLTSFGDAIKNMDNLKGAFAKLSELHCDKLHVDPENFRLLGNVLVIILATHFGKDFTPEVQVAWQKLVSGVAIALAHKYH
HBE1_marmoset        MVHFTAEEKAAITSLWGKMNVEEAGGEALGRLLVVYPWTQRFFDNFGNLSSPSAILGNPKVKAHGKKVLTSFGDAIKNMDNLKTTFAKLSELHCDKLHVDPENFKLLGNVLVIILATHFGKEFTPEVQAAWQKLVSAVAIALGHKYH
HBE1_macaque         MVHFTAEEKAAVTSLWSKMNVEETGGEALGRLLVVYPWTQRFFDSFGNLSSPSAILGNPKVKAHGKKVLTSFGDAIKNMDNLKITFAKLSELHCDKLHVDPENFKLLGNVMVIILATHFGKEFTPEVQAAWQKLVSAVAIALAHKYH
HBE1_human           MVHFTAEEKAAVTSLWSKMNVEEAGGEALGRLLVVYPWTQRFFDSFGNLSSPSAILGNPKVKAHGKKVLTSFGDAIKNMDNLKPAFAKLSELHCDKLHVDPENFKLLGNVMVIILATHFGKEFTPEVQAAWQKLVSAVAIALAHKYH
HBE1_gorilla         MVHFTAEEKAAVTSLWSKMNVEEAGGEALGRLLVVYPWTQRFFDSFGNLSSPSAILGNPKVKAHGKKVLTSFGDAIKNMDNLKPAFAKLSELHCDKLHVDPENFKLLGNVMVIILATHFGKEFTPEVQAAWQKLVSAVAIALAHKYH
HBE1_gibbon          MVHFTAEEKAAVTSLWNKMNVEEAGGEALGRLLVVYPWTQRFFDSFGNLSSPSAILGNPKVKAHGKKVLTSFGDAIKNMDNLKTTFAKLSELHCDKLHVDPENFKLLGNVMVIILATHFGKEFTPEVQAAWQKLVSAVAIALAHKYH
HBE1_orangutan       MVHFTAEEKAAVTSLWSKMNVEEAGGEALGRLLVVYPWTQRFFDSFGNLSSPSAILGNPKVKAHGKKVLTSFGDAIKNMDNLKTTFAKLSELHCDKLHVDPENFKLLGNVMVIILATHFGKEFTPEVQAAWQKLVSAVAIALAHKYH
HBE1_pika            MVHFTPEEKCIISKQWSQVNVEETGGEALGRLLVVYPWTQRFFDSFGNLSSSSAIMGNPKVKAHGKKVLTSFGEAIKNMDNLKTAFAKLSELHCDKLHVDPENFKLLGNVLLIVLATHFGKEFSPEVQAAWQKLVSGVAIALAHKYH
HBE1_rabbit          MVHFTPEEKCIISKQWGQVNIDETGGEALGRLLVVYPWTQRFFDNFGNLSSSSAIMGNPKVKAHGKKVLTSFGDAIKNMDNLKGAFAKLSELHCDKLHVDPENFKLLGNVLLIVLATHFGKEFTPEVQAAWQKLVSGVAIALAHKYH
HBE1_guinea_pig      MVHFTTEEKSIINALWGKVNVEETGGETLGRLLVVYPWTQRFFDNFGNLSSPSAIMGNPKVKAHGKKVLTSFGEAIKNLDNLKGTFAKLSELHCDKLHVDPENFRLLGNVLLIVLATHFGKEFTPDVQAAWQKLVSGVAIALAHKYH
HBG_elephant         MVHFTAEEKAAITSLWGQVNVEETGGEALGRLLVVYPWTQRFFDTFGNLSSASAIMGNPRVKAHGKKVLTSFGDAVKNLDNLKGTFAKLSELHCDKLHVDPENFRLLGNVLVIVLANHFGKEFTPQVQAAWQKMVTGVANALAYKYH
HBGA_bushbaby        MVHFTAEEKAIIMSLWGKVNIEEAGGEALGRLLVVYPWTQRFFDTFGNLSSASAIMGNPKVKAHGKKVLTSFGEAVKNMDNLKGAFAKLSELHCDKLHVDPENFKLLGNVMVIILATHFGKEFTPDVQAAWQKLVSGVATALAHKYH
HBE1_cow             MVHFTAEEKAAITGLWGKVNVEEAGGEALGRLLVVYPWTQRFFDSFGNLSSASAIMGNPKVKAHGKKVLTSFGEAIKNLDNLKGAFAKLSELHCDKLHVDPENFRLLGNVIVIILATHFGREFTPDVQAAWQKLVSGVATALAHKYH
HBE_bat              MVHFTGEEKAVIANMWGKMNVEEAGGEALGRLLVVYPWTQRFFDSFGNLSSSSAIMGNPKVKAHGKKVLTSFGDAIKNMDNLKGTFAKLSELHCDKLHVDPENFRLLGNVLVIILASHFGKEFTPEVQAAWQKLVASVANALAHKYH
HBE1_pig             MVHFTAEEKSVITGLWGKVNVEETGGEAVGRLLVVYPWTQRFFDSFGNMSSPSAIMGNPKVKAHGKKVLTSFGDAVKNMDNLKGTFAKLSELHCDKLHVDPENFRLLGNMIVIILASHFGGEFTPEVQAAWQKLVAGVATALAHKYH
HBE1_microbat        MVHFTGEEKAAVTNVWSKVNVEEAGGEVLGRLLVVYPWTQRFFDNFGNLSSSSAIMGNPKVKAHGKKVLTSFGDAIKNMDNLKSAFAKLSELHCDKLHVDPENFRLLGNVMVIVLASHFGKEFTPDVQAAWQKLVAGVANAMAHKYH
HBE1_elephant        MVHFTAEEKSAVTSLWGKVNVEEAGGEALGRLLVVYPWTQRFFDSFGNLSSASAIMGNPKVKAHGKKVLTSFGDAVKNMDNLKGAFAKLSELHCDKLHVDPENFRLLGNVLVIILASHFGREFTPDMQAAWQKLVSGVANALSHKYH
N/A-1_megabat        MVHFTGEEKAAITNVWGKVNVEEAGGEALGRL-GVYPWTQRFFDNFGNLSSASAIMGNPKVKAHGKKVLTSFGDAIKNLDNLKSAFAKLSELHCDKLHVDPENFRLLGNVLVIILASHFGKEFTPDVQAAWQKLVLGVANALAHKYH
HBE1_dolphin         MVHFTAEEKAAITGLWGRVNVEEAGGEALGRLLVVYPWTQRFFDNFGNLSSPSAIMGNPKVKAHGKKVLTSFGDAVKNMDNLKGAFAKLSELHCDKLHVDPENFRLLGNVLVIILASHFGREFTPELQAAWQKLVAGVATALAHKYH
HBE_dog              MVHFSAEEKAAITSLWGKVNVEDAGGEALGRLLVVYPWTQRFFDNFGNLSSASAIMGNPKVKAHGKKVLTSFGDAVKNMDNLKGTFAKLSELHCDKLHVDPENFRLLGNVLVIILASHFGKEFTPDVQAAWQKLVAGVATALAHKYH
HBE1_horse           MVHFTAEEKAAITNAWGKVNVEEAGGEALGRLLVVYPWTQRFFDNFGNLSSSSAIMGNPKVKAHGKKVLTSFGDAVKNMDNLKAAFAKLSELHCDKLHVDPENFRLLGNVLVIILASHFGKEFTPDVQAAWQKLVSGVANALAHKYH
N/A_cat              MVHFTAEEKAAITNLWGKVNVEEAGGEALGRLLVVYPWTQRFFDNFGNLSSTSAIMGNPKVKAHGKKVLTSFGDAIKNMDNLKGAFAKLSELHCDKLHVDPENFRLLGNVLVIILASHFGKEFTPDMQAMWQKLVAGVATALAHKYH
```

B)

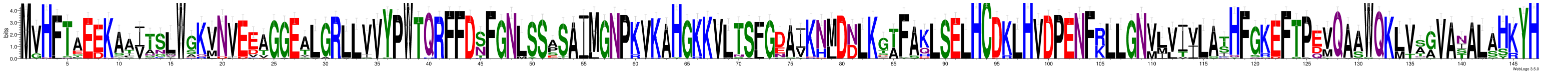

Additional file 4. Weblogo analysis of embryonic beta globin proteins. (A) Proteins from diverse placental mammals were taken from Treefam (see Methods) for comparison of the canine proteins to others and their consensus. (B) Weblogo is a graphic representation of amino acid frequency at each position. Here the Weblogo is aligned with the multiple sequence alignment of the same proteins above it.
