# Supplementary material for: Comparative genomics of canine hemoglobin genes reveals primacy of beta subunit delta in adult carnivores
Source: BMC Genomics. 2017 Feb 8;18:141. doi: 10.1186/s12864-017-3513-0 (PMC5299747; doi:10.1186/s12864-017-3513-0)
Supplement: Additional file 5: — A) Sequence alignment of adult β-globins from model placental mammal genomes, and B) consensus sequence logo from those adult β-globins. (PDF 1065 kb) [file 12864_2017_3513_MOESM5_ESM.pdf]

A)

```
                 10        20        30        40        50        60        70        80        90        100       110       120       130       140
N/A_guinea_pig   MVHLTAAEKSAILDLWGKVNVGEIGAEALGRLLVVYPWTQRFFEKFGDLSSASAIMSNAHVKSHGAKVLASFSEGLKHLEDLKGTFAKLSELHCDKLHVDPENFRLLGNMIVIALAHHHPSEFTPCTQAAFQKVTAGVANALAHKYH
N/A_kangaroo_rat MVHLSGEEKAAVAAVWSKVNVDSAGAEALGRLLCVYPWTQRFFDSFGDLSSADAIMNNPKVKAHGKKVLHSFDDGLHHLDNLKATYASLSELHCDKLHVDPENFRLLGNVIVVVLSRNLGKDFTPVAQAAFQKVVSGVATALAHKYH
HBB1_armadillo   MVNLTSVEKSAVTGLWRKVDVEDCGGEALGRLLVVYPWTQRFFESFGDLSTPAAVFANAKVKAHGKKVLTSFGEGMNHLDNLKGTFAKLSELHCDKLHVDPENFRLLGNMLVVVMARHFGKEFDWHMHACFQRVVAGVANALAHKYH
HBB2_armadillo   MVNLTSDEKTAVLALWNKVDVEDCGGEALGRLLVVYPWTQRFFESFGDLSTPAAVFANAKVKAHGKKVLTSFGEGMNHLDNLKGTFAKLSELHCDKLHVDPENFKLLGNMLVVVLARHFGKEFDWHMHACFQKVVAGVANALAHKYH
HBD_elephant     MVNLTAAEKTQVTNLWGKVNVKELGGEALSRLLVVYPWTRRFFEHFGDLSTAEAVLHNAKVLAHGEKVLTSFGEGLKHLDNLKGTFADLSELHCDKLHVDPENFRLLGNVLVIVLARHFGKEFTPDVQAAYEKVVAGVANALAHKYH
HBBD_elephant    MVNLTAAEKTQVTNLWGKVNVKELGGEALSRLLVVYPWTRRFFEHFGDLSTAEAVLHNAKVLAHGEKVLTSFGEGLKHLDNLKGTFADLSELHCDKLHVDPENFRLLGNVLVIVLARHFGKEFTPDVQAAYEKVVAGVANALAHKYH
N/A-1_sloth      MVHLTDDEKAAVS-ALGKVDVEEVGGEALGRLLVVYPWTQRFFEHFGDLSTADAVFSNPKVKAHGKKVLTSFSDGLKHLDNLKDTYAQLSELHCDKLHVDPENFRLLGNIVVIVLAHRFGKEFTPELQASYQKVVTGVANALAHKYH
N/A-2_sloth      MVHLTDDEKAAISALWGKVDVEQVGGEALGRLLVVYPWTQRFFEHFGDLSTADAVFSNPKVKAHGKKVLTSFSDGLKHLDNLKDTYAHLSELHCDKLHVDPENFKLLGNVLVIVLAHHFGKEFTVPLQAAYQKVVTGVANALAHRYH
HBB1_mouse       MVHLTDAEKAAVSCLWGKVNSDEVGGEALGRLLVVYPWTQRYFDSFGDLSSASAIMGNAKVKAHGKKVITAFNDGLNHLDSLKGTFASLSELHCDKLHVDPENFRLLGNMIVIVLGHHLGKDFTPAAQAAFQKVVAGVATALAHKYH
HBB2_mouse       MVHLTDAEKSAVSCLWAKVNPDEVGGEALGRLLVVYPWTQRYFDSFGDLSSASAIMGNPKVKAHGKKVITAFNEGLKNLDNLKGTFASLSELHCDKLHVDPENFRLLGNAIVIVLGHHLGKDFTPAAQAAFQKVVAGVATALAHKYH
HBB2_horse       MVQLSGEEKTAILALWGKVNEEEIGGEALGRLLIVYPWTQRFFDSFGHLTTSAAVTGNPRVKAHGKKVLHSFGEGVHHLDNLKDTFAQLSELHCDKLHLDPENFILLGNVLDVVLAYHFGKEFTPELQASYQKAVSGGAHALAEHYH
HBB_pig          MVHLSAEEKEAVLGLWGKVNVDEVGGEALGRLLVVYPWTQRFFESFGDLSNADAVMGNPKVKAHGKKVLQSFSDGLKHLDNLKGTFAKLSELHCDQLHVDPENFRLLGNVIVVVLARRLGHDFNPDVQAAFQKVVAGVANALAHKYH
HBB_alpaca       MVNLSGDEKNAVHGLWSKVKVDEVGGEALGRLLVVYPWTRRFFESFGDLSTADAVMNNPKVKAHGSKVLNSFGDGLSHLDNLKGTYAKLSELHCDKLHVDPENFRLLGNVLVVVLARHFGKEFTPDLQAAYQKVVAGVANALAHRYH
HBB1_horse       MVQLSGEEKAAVLALWDKVNEEEVGGEALGRLLVVYPWTQRFFDSFGDLSNPGAVMGNPKVKAHGKKVLHSFGEGVHHLDNLKGTFAALSELHCDKLHVDPENFRLLGNVLVVVLARHFGKDFTPELQASYQKVVAGVANALAHKYH
N/A-2_microbat   MVHLTADEKAAVSGLWGKVNVDEVGGEALGRLLVVYPWTQRFFTSFGDLSNAAAVMGNSKVKAHGKKVLNSFGEGLKNVDNLKGTFASLSELHCDKLHVDPENFKLLGNVLVVVLARHFGKEFTPQVQGAFQKLALGVATALAHKYH
HBB_dolphin      MVHLTGEEKSAVTALWGKVNVEEVGGEALGRLLVVYPWTQRFFESFGDLSTADAVMKNPNVKKHGQKVLASFGEGLKHLDDLKGTFAALSELHCDKLHVDPENFRLLGNVLVVVLARHFGKEFTPELQSAYQKVVAGVATALAHKYH
N/A_dolphin      MVHLTGEEKSAVTALWGKVNVEEVGGEALGRLLVVYPWTQRFFESFGDLSTADAVMKNPNVKKHGQKVLASFGEGLKHLDDLKGTFAALSELHCDKLHVDPENFRLLGNVLVVVLARHFGKEFTPELQAAYQKVAPGVANALAHNYH
HBB_tarsier      MVHLTADEKAAVTALWSKVNVEDVGGEALGRLLVVYPWTQRFFDSFGDLSTPAAVMSNAKVKAHGKKVLNAFSDGMAHLDNLKGTFAKLSELHCDKLHVDPENFRLLGNVLVCVLAHHFGKQFTPQLQAAYQKVVAGVAAALAHKYH
HBB_bushbaby     MVHLTPDEKNAVCALWGKVNVEEVGGEALGRLLVVYPWTQRFFDSFGDLSSPSAVMGNPKVKAHGKKVLSAFSEGLNHLDNLKGTFAKLSELHCDKLHVDPENFRLLGNVLVVVLAHHFGKDFTPQVQAAYQKVVAGVATALAHKYH
HBD_bushbaby     MVHLTPDEKNAVCALWGKVNVEEVGGEALGRLLVVYPWTQRFFDSFGDLSSPSAVMGNPKVKAHGKKVLSAFSEGLNHLDNLKGTFAKLSELHCDKLHVDPENFRLLGNVLVVVLAHHFGKDFTPEVQAAYQKVVAGVATALAHKYH
HBB_rabbit       MVHLSSEEKSAVTALWGKVNVEEVGGEALGRLLVVYPWTQRFFESFGDLSSAHAVMSNPKVKAHGKKVLAAFSEGLNHLDNLKGTFAKLSELHCDKLHVDPENFRLLGNVLVVVLSHHFGKEFTPQVQAAYQKVVAGVANALAHKYH
HBD_human        MVHLTPEEKTAVNALWGKVNVDAVGGEALGRLLVVYPWTQRFFESFGDLSSPDAVMGNPKVKAHGKKVLGAFSDGLAHLDNLKGTFSQLSELHCDKLHVDPENFRLLGNVLVCVLARNFGKEFTPQMQAAYQKVVAGVANALAHKYH
HBB_macaque      MVHLTPEEKTAVTTLWGKVNVDEVGGEALGRLLVVYPWTQRFFDSFGDLSSPDAVMGNPKVKAHGKKVLGAFSDGLNHLDNLKGTFAQLSELHCDKLHVDPENFKLLGNVLVCVLAHHFGKEFTPQVQAAYQKVVAGVANALAHKYH
HBB_human        MVHLTPEEKSAVTALWGKVNVDEVGGEALGRLLVVYPWTQRFFESFGDLSTPDAVMGNPKVKAHGKKVLGAFSDGLAHLDNLKGTFATLSELHCDKLHVDPENFRLLGNVLVCVLAHHFGKEFTPPVQAAYQKVVAGVANALAHKYH
HBB_orangutan    MVHLTPEEKSAVTALWGKVNVDEVGGEALGRLLVVYPWTQRFFESFGDLSTPDAVMGNPKVKAHGKKVLGAFSDGLAHLDNLKGTFAKLSELHCDKLHVDPENFRLLGNVLVCVLAHHFGKEFTPQVQAAYQKVVAGVANALAHKYH
HBBL_ferret      MVHLSAEEKAAVSSLQGKVKVDVVGGETLGRLLVVYPWTQRFFDSFGDLSSPDAIMSNGKVKAHGKKVVNSLINGLKNLDNLKGAFAKLSELHCDKLHVDPENFKLLGNVMVCVLARHFGKEFTPQVQAVFQKVLAGVANALAHKYH
HBB_ferret       MVHLTGEEKAAVTALWGKVNVDEVGGETLGRLLVVYPWTQRFFDSFGDLSSPDAVMSNPKVKAHGKKVLNSFSEGLKNLDNLKGTFAKLSELHCDKLHVDPENFKLLGNVLVCVLAHHFGKEFTPQVQAAYQKVVAGVANALAHKYH
HBB_panda        MVHLTGEEKAAVTGLWSKVNVDEVGGEALGRLLVVYPWTQRFFDSFGDLSTPDAVMNNPKVKAHGKKVLNSFSEGLKNLDNLKGTFAKLSELHCDKLHVDPENFKLLGNVLVCVLAHHFGKEFTPQVQAAYQKVVAGVANALAHKYH
HBB_dog          MVHLTAEEKSLISSMWGKVNVDEVGGEALGRLLIVYPWTQRFFDSFGDLSTPDAVMSNAKVKAHGKKVLNSFSDGLKNLDNLKGTFAKLSELHCDKLHVDPENFKLLGNVLVCVLARHFGREFTPLAQAAYQKVVAGVANALAHKYH
HBD12_dog        MVHLTAEEKSLVSGLWGKVNVDEVGGEALGRLLIVYPWTQRFFDSFGDLSTPDAVMSNAKVKAHGKKVLNSFSDGLKNLDNLKGTFAKLSELHCDKLHVDPENFKLLGNVLVCVLAHHFGKEFTPQVQAAYQKVVAGVANALAHKYH
HBD1_shrew       MVHLSGEEKGVVTGLWGKVNVDEVGGEALGRLLVVYPWTQRFFDSFGDLSSASAIMGNPKVKAHGKKVLQSLGDGINNLDNLKGTFAKLSELHCDKLHVDPENFRLLGNVLVCVLARHFGKEFTPSVQAAFQKMVAGVAAALAHKYH
HBD2_shrew       MVHLSGEEKGVVTGLWGKVNVDEVGGEALGRLLVVYPWTQRFFDSFGDLSSASAIMGNPKVKAHGKKVLQSLGDGINNLDNLKGTFAKLSELHCDKLHVDPENFRLLGNVLVCVLARHFGKEFTPSVQAAFQKMVAGVAAALAHKYH
N/A-2_megabat    MVHLSGEEKAAVTGLWGKVKVDEVGGEALGRLLVVYPWTQRFFDSFGDLSSASAVMGNPKVKAHGKKVLDSFSEGLQHLDNLKGTFAKLSELHCDKLHVDPENFRLLGNVLVCVLARHFGKEFTPQVQAAYQKVVAGVATALAHKYH
HBB_bat          MVHLTGEEKGIVTGLWGKVNVDEVGGEALGRLLVVYPWTQRFFDSFGDLSSAAAVMGNAKVKAHGKKVLDSFSEGLKNLDNLKGTFAKLSELHCDKLHVDPENFRLLGNVLVCVLARNFGKEFTPQVQAAYQKVVVGVATALAHKYH
HBD_bat          MVHLTGEEKGIVTGLWGKVNVDEVGGEALGRLLVVYPWTQRFFDSFGDLSSAAAVMGNAKVKAHGKKVLDSFSNGMKNLDNLKGTFAKLSELHCDKLHVDPENFRLLGNVLVCVLARNFGKDFTPQVQAAYQKVVAGVATALAHKYH
```

B)

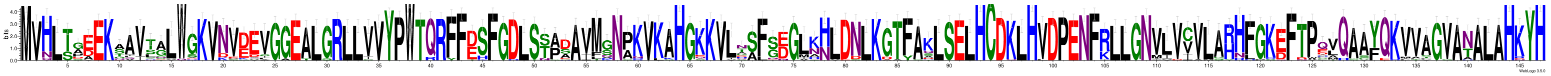

Additional file 5. Weblogo analysis of adult beta globin proteins. (A) Proteins from diverse placental mammals were taken from Treefam (see Methods) for comparison of the canine proteins to others and their consensus. (B) Weblogo is a graphic representation of amino acid frequency at each position. Here the Weblogo is aligned with the multiple sequence alignment of the same proteins above it.
